# *In vivo* zebrafish morphogenesis shows Cyp26b1 promotes tendon condensation and musculoskeletal patterning in the embryonic jaw

**DOI:** 10.1371/journal.pgen.1007112

**Published:** 2017-12-11

**Authors:** Patrick D. McGurk, Mary E. Swartz, Jessica W. Chen, Jenna L. Galloway, Johann K. Eberhart

**Affiliations:** 1 Department of Molecular Biosciences, University of Texas at Austin, Austin, TX, United States of America; 2 Center for Regenerative Medicine, Harvard Stem Cell Institute, Department of Orthopaedic Surgery, Massachusetts General Hospital, Boston, MA, United States of America; 3 Department of Genetics, Harvard Medical School, Cambridge, MA, United States of America; University of California, San Francisco, UNITED STATES

## Abstract

Integrated development of diverse tissues gives rise to a functional, mobile vertebrate musculoskeletal system. However, the genetics and cellular interactions that drive the integration of muscle, tendon, and skeleton are poorly understood. In the vertebrate head, neural crest cells, from which cranial tendons derive, pattern developing muscles just as tendons have been shown to in limb and trunk tissue, yet the mechanisms of this patterning are unknown. From a forward genetic screen, we determined that *cyp26b1* is critical for musculoskeletal integration in the ventral pharyngeal arches, particularly in the mandibulohyoid junction where first and second arch muscles interconnect. Using time-lapse confocal analyses, we detail musculoskeletal integration in wild-type and *cyp26b1* mutant zebrafish. In wild-type fish, tenoblasts are present in apposition to elongating muscles and condense in discrete muscle attachment sites. In the absence of *cyp26b1*, tenoblasts are generated in normal numbers but fail to condense into nascent tendons within the ventral arches and, subsequently, muscles project into ectopic locales. These ectopic muscle fibers eventually associate with ectopic tendon marker expression. Genetic mosaic analysis demonstrates that neural crest cells require Cyp26b1 function for proper musculoskeletal development. Using an inhibitor, we find that Cyp26 function is required in a short time window that overlaps the dynamic window of tenoblast condensation. However, *cyp26b1* expression is largely restricted to regions between tenoblast condensations during this time. Our results suggest that degradation of RA by this previously undescribed population of neural crest cells is critical to promote condensation of adjacent *scxa*-expressing tenoblasts and that these condensations are subsequently required for proper musculoskeletal integration.

## Author summary

Mobility requires that muscles form appropriate attachments via tendon. The genes regulating this attachment are poorly understood. This is especially true in the head where mesoderm and neural crest cells generate muscle and tendon, respectively. We show that the gene *cyp26b1* is critical for muscle tendon attachment in a specific region of the developing head. The movement of tendon generating cells into mature tendons requires *cyp26b1*. Loss of *cyp26b1* disrupts these movements and muscles subsequently extend into inappropriate areas. Cyp26b1 appears to be required in neural crest cells but not the tendon generating cells. Collectively, our work provides insight into the genetic and cellular mechanisms that attach muscle and tendon.

## Introduction

The movements and functions of the human head depend on 150 individual muscles, and loss of tendon or of tendon-muscle interactions has been implicated in human craniofacial syndromes with muscle defects. Vertebrate craniofacial development is a complex process involving communication between muscles, tendons, cartilages and surrounding tissues. Much of this communication occurs in transient, reiterated, pharyngeal arches. Within each pharyngeal arch are neural crest cells that form a specific set of skeletal elements and mesoderm cells that form a specific set of muscles[1]. The developmental origins of the various cranial tendons remain unclear, save that they derive from the neural crest [2, 3]. Given that neural crest cells control the patterning of cranial muscles [4, 5], it is likely that cranial tendons provide the positional cues needed for this patterning. We know little, however, about the genetics and cellular interactions underlying the mechanisms of development at the myotendinous junction.

We have a limited number of tendon disease models from which to gain an understanding of tendon functions in development. Muscle elements form and elongate after surgical removal of tendon primordia in the developing avian hindlimb, but in these limbs, muscle fibers are ectopically localized [6]. This result suggests that tendons restrict the patterning of limb musculature. Genetic disruption of *Scx* in mice causes defects in force-transmitting tendons in the trunk, limbs, and tail [7]. However, Scx function is not necessary for tendon to develop and form functional muscle attachments in mutant mice. Scx is also not sufficient for tendon development because tendon progenitors (tenoblasts) are specified in the pharyngeal arches but eventually disappear in muscle-less mutant models [2, 8]. These findings demonstrate that in the head, unlike in the somites [9], specification of tendons is independent of muscle. However, in all tendon populations muscle provides mechanical stimulation that promotes growth factor signaling and *Scx* expression, and muscle contraction is as necessary as the muscle tissue itself for tendon differentiation [10]. Thus, to study tendon functions it will be essential to discover genetic models in which muscle differentiation is normal but muscle attachments are defective.

**Fig 1:**
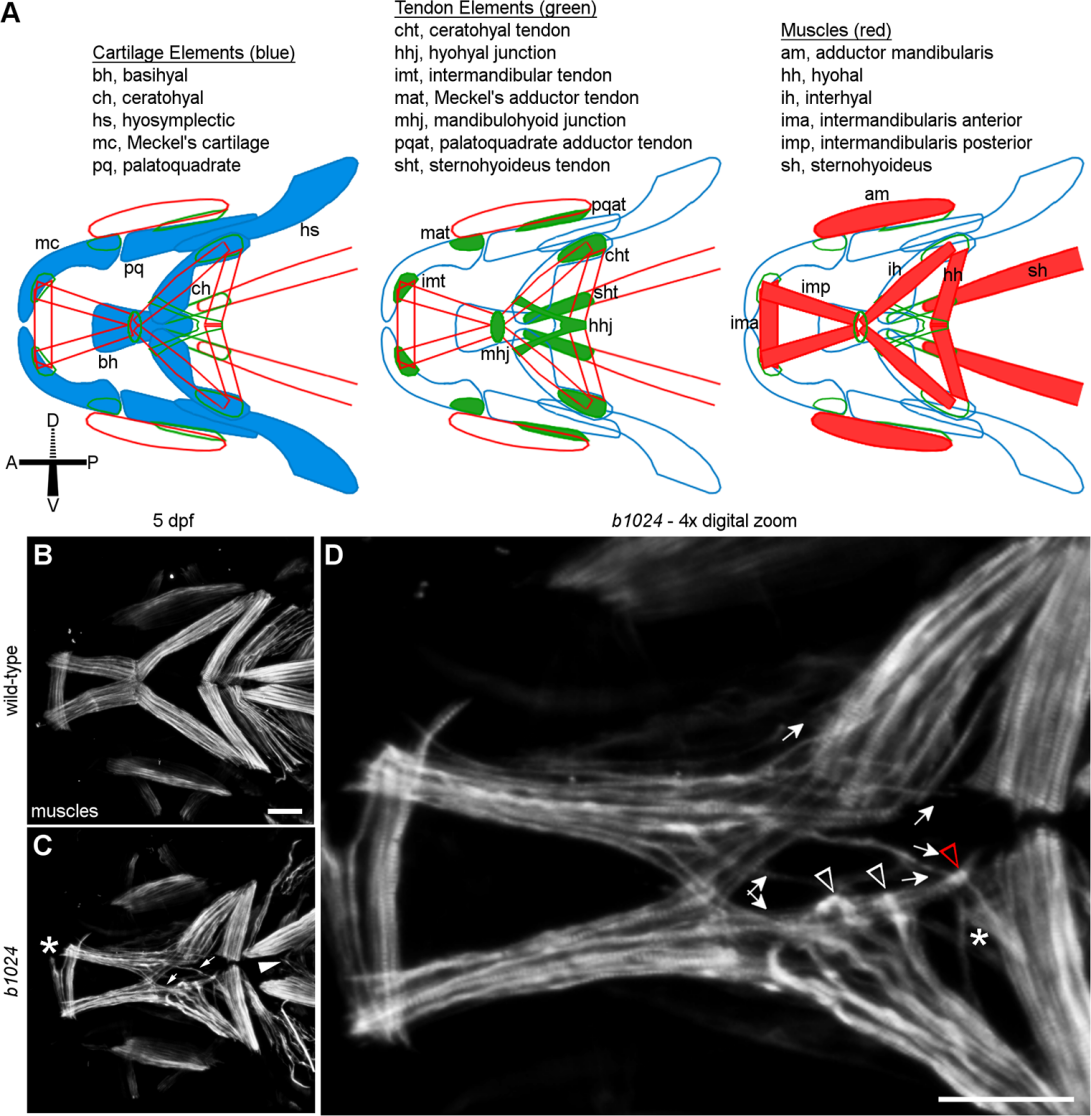
Homozygous carriers of *b1024* display second pharyngeal arch lower jaw muscle defects. (A) Schematic of connective tissues in the larval zebrafish jaw, modified from Chen and Galloway, 2014. (B, C) Ventral craniofacial muscles in wild-type and *b1024* embryos. (C) Muscle fibers from an intermandibularis posterior muscle extend past the mandibulohyoid junction and insert alongside the adjacent interhyal muscle or at the hyohyal junction (arrows). Muscle fibers apparently from the sternohyoideus are present in the ventral midline of mutants (arrowhead). Ectopic muscle fibers split off from the intermandibularis anterior muscle in a small percentage of mutants (asterisk). (D) 4x digital zoom of a single Z slice from confocal image in C. Arrows show ectopic paths of intermandibularis posterior muscle fibers. Arrowheads show ectopic midline attachments of interhyal muscle fibers. A three-way junction (red arrowhead in D) forms between ectopic intermandibularis posterior, interhyal, and hyohyal (asterisk in D) muscle fibers. All images ventral view, anterior to the left. Scale bars = 50 μm.

**Fig 2:**
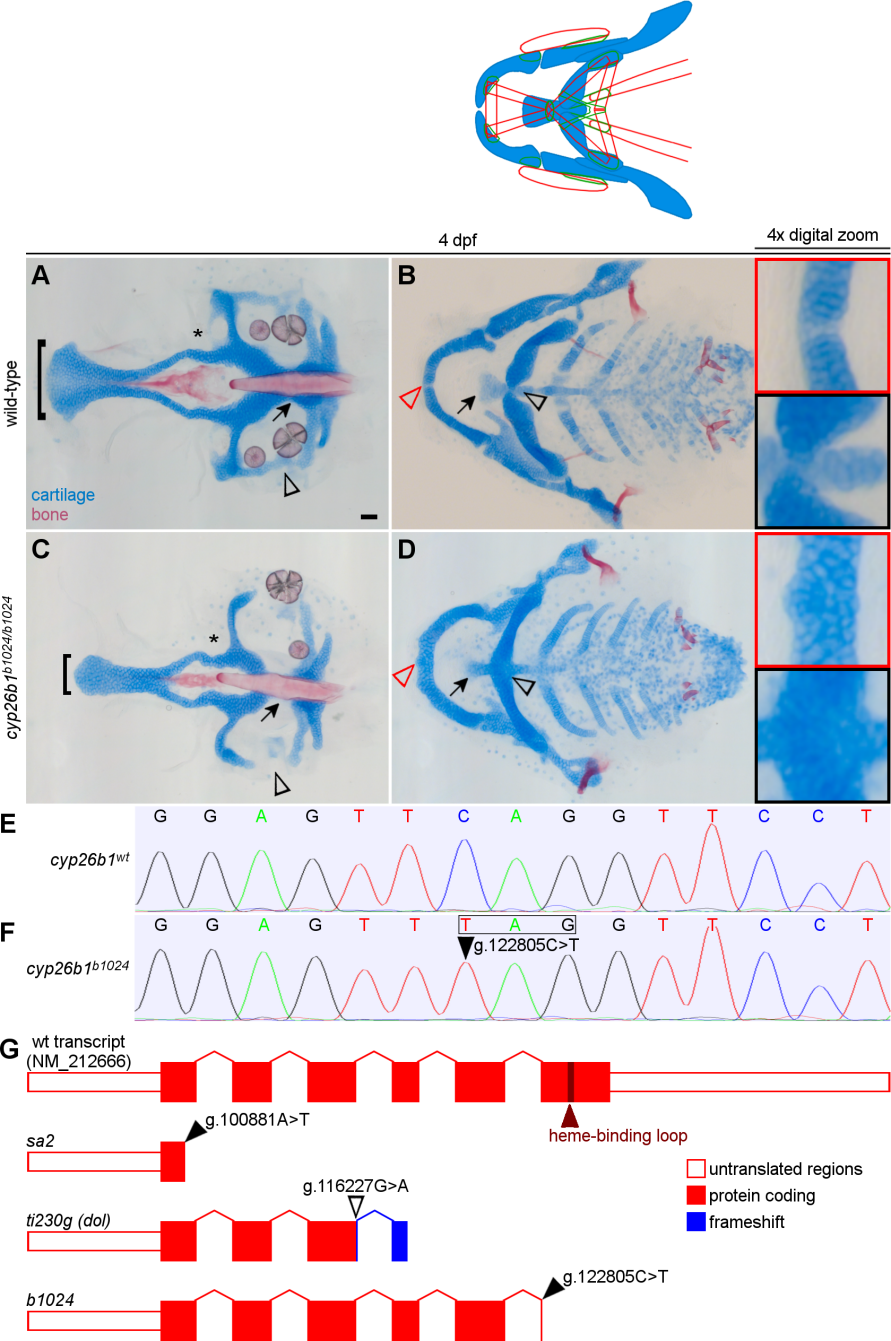
The *b1024* lesion is a nonsense mutation in *cyp26b1* that causes skeletal defects. (A-B) Wild-type head skeleton stained with Alcian Blue and Alizarin Red dyes. (C) In *b1024* mutants the ethmoid plate (brackets in A,C) and parasphenoid bone are narrow and the parachordal (arrows in A,C), basicapsular commissure (arrowheads in A,C), and lateral commissure (asterisks in A,C) cartilages of the posterior neurocranium are hypoplastic. (D) The midline symphysis of Meckel’s (red arrowheads and red insets in B,D) and ceratohyal (black arrowheads and black insets in B,D) cartilages are absent in *b1024* homozygotes, fusing the left and right cartilage elements, and the basihyal cartilage (arrows in B,D) is hypoplastic. (E-F) A single base substitution in *cyp26b1* (arrowhead) results in an in-frame stop codon (box). (G) *b1024* causes a later truncation of *cyp26b1* than previously characterized alleles, but still eliminates essential components of the cytochrome P450 enzyme domain encoded in exon 6. All images ventral view, anterior to the left. Scale bars = 50 μm.

Zebrafish is an ideal system for forward genetic analysis and has a complex but well-characterized craniofacial musculoskeletal system (Fig 1A; [1, 2]). In the zebrafish head, the more ventral portion of the first and second pharyngeal arches gives rise to the lower jaw and its supports [1]. Seven muscles extend across the ventral surface of both arches in an hourglass-like shape whose center sits at the anterior tip of the second arch. This central attachment, or mandibulohyoid junction, is comprised of tendon and the ends of four of these muscles, two intermandibularis posterior muscles originating from the first arch and two interhyal muscles from the second arch [2]. Similarly, tendon develops between the two hyohyal muscles at the posterior of the second arch. At the remaining attachments, tendon connects muscle to cartilage. All of these tendons express the zebrafish *Scx* ortholog, *scxa*, and *xirp2a*, whose expression requires muscle [2].

Thus far, there are no *in vivo* models detailing the morphogenetic dynamics of tendon and muscle. Few genes have yet been determined necessary for tendon development, though tendon function is impaired by genetic knockdown of *Scx* or tendon extracellular matrix components [7, 11]. In a zebrafish forward genetic screen for craniofacial mutants, we discovered a novel allele of *cyp26b1* with a craniofacial skeletal phenotype consistent with previously described loss-of-function alleles [12–14]. Larvae homozygous for this mutation also fail to close their jaws. Our *in vivo* analysis of wild-type and *cyp26b1* mutant embryos revealed essential steps in the morphogenesis of the ventral first- and second-arch muscles responsible for jaw movement. We show that neural crest cells require the retinoic-acid-catabolizing enzyme Cyp26b1 to pattern jaw muscle attachments in the first and second pharyngeal arches. We propose that *cyp26b1* expression is required in a mass of non-tendon neural crest that separates adjacent tenoblast populations and promotes tendon condensation necessary for normal muscle patterning.

## Results

### Cranial muscles have defective attachments in *b1024* mutants

To investigate the mechanisms responsible for integrating the cranial musculoskeletal system, we analyzed the muscle phenotypes of craniofacial mutants, identified in an ENU-based forward genetic screen in zebrafish. We identified the *b1024* allele, whose homozygous mutant offspring had defects in ventral first- and second-arch jaw muscles (see Fig 1A for schematics of these jaw muscles and associated cartilages and tendons). Though these muscles mostly retained their stereotypic pattern in mutants, muscle fibers often split from the main mass to project and terminate ectopically (Fig 1B).

Not all of the jaw muscles were equally defective in *b1024* homozygotes (See Table 1 for quantification). Most commonly, the intermandibularis posterior muscles (Fig 1C, arrows, 76% of mutants n = 13/17) and/or interhyal muscles (Fig 1D, white arrowheads, 71% of mutants, n = 12/17) sent ectopic projections into the medial second pharyngeal arch. Intermandibularis posterior muscle fibers overextended toward the midline and terminated at the junction between hyohyal muscles, on the interhyal, or at other positions on the medial second arch surface (Fig 1C and 1D, arrows). Muscle fibers split off from the hyohyal muscles (almost always toward other ectopic muscle fibers) in 24% of mutants (asterisk in Fig 1D, n = 4/17). The intermandibularis anterior muscle fibers split and formed ectopic attachments in 12% of mutants (asterisk in Fig 1C, n = 2/17). The sternohyoideus muscle, which also attaches to this region, projected across the midline in 84% of mutants (arrowhead in Fig 1C, n = 16/19). The disruption to muscle attachment appears highly specific to the ventral interface between the first and second arch as the more dorsal muscles in this region of the head appeared normal in *b1024* mutants (S1 Fig). These results indicate craniofacial musculoskeletal development is, at least somewhat, modular and that the *b1024* mutation disrupts musculoskeletal connections between the first and second pharyngeal arch midline.

**Table 1:** Quantification of observed phenotypes.

Phenotype	wt	*cyp26b1*^*-/-*^
IMP overextension	3% (2/49)	76% (13/17)
ectopic IH fibers	18% (9/49) *all minor	71% (12/17) *all severe
HH no midline connection	0% (0/49)	88% (15/17)
HH ectopic fibers	10% (5/49) *all minor	24% (4/17) *all severe
IMA ectopic fibers	8% (4/49)	12% (2/17)
SH muscle fibers crossing midline	0% (0/11)	84% (16/19)

### A novel mutation in *cyp26b1* causes craniofacial musculoskeletal defects in *b1024*

In addition to muscle defects, *b1024* mutants had midline skeletal defects (Fig 2A–2D). Homozygous zebrafish had a narrow ethmoid plate and parasphenoid bone (Fig 2C). In the posterior neurocranium, mutants displayed a gap in the parachordal cartilages (arrows in Fig 2A and 2C), medial to the ear, and a gap between the anterior and posterior basicapsular cartilages (arrowheads in Fig 2A and 2C), lateral to the ear (Fig 2C). In the viscerocranium of *b1024* mutants, the ventral cartilage elements of the first and second pharyngeal arches, Meckel’s cartilages and ceratohyal cartilages, respectively, were fused in the midline (arrowheads and insets in Fig 2B and 2D). The anterior end of the basihyal cartilage was narrow and hypoplastic compared to wild-type larvae, similar to the ethmoid plate defect (arrows in Fig 2B and 2D). Collectively, these results demonstrated that *b1024* mutants have prominent musculoskeletal defects largely localized to the ventral midline.

To identify the genetic lesion in *b1024*, we performed linkage analysis with simple sequence length polymorphisms. We genetically mapped the *b1024* lesion to an interval of chromosome 7 between z8889 and z1239 containing *cyp26b1*. While muscle phenotypes have not been previously analyzed in zebrafish *cyp26b1* mutants, the *b1024* skeletal phenotype fit with previous descriptions of *cyp26b1* mutants [13]. We discovered a single base substitution (C > T) seven bases into exon 6 (Fig 2E–2G, RefSeq NM_212666). This variant creates an early stop codon (p.Gln384*, RefSeq NP_997831) that is predicted to truncate the last 128, of 511 total, amino acids of Cyp26b1. A single cytochrome P450 superfamily domain comprises most of the protein sequence. This lesion in *cyp26b1* truncates 23% of the P450 domain, including the highly conserved heme-binding loop in exon 6, which is required for enzymatic activity [15]. We conclude that *b1024* is a recessive loss-of-function, likely null, allele of *cyp26b1* like *sa2* [14] and *ti230g* [12].

### Loss of Cyp26b1 function causes abnormal jaw muscle morphogenesis

**Fig 3:**
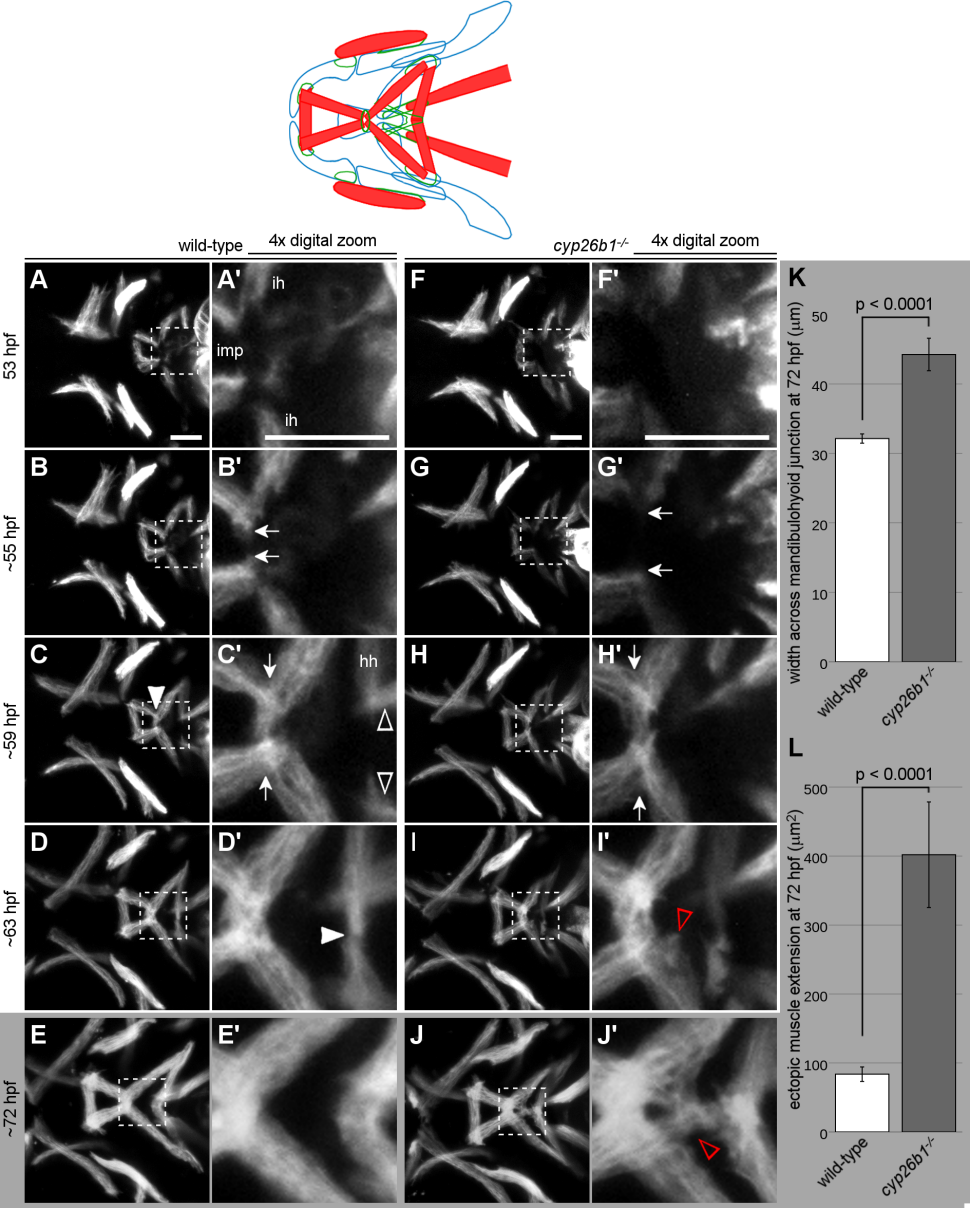
Loss of Cyp26b1 function disrupts morphogenesis of the mandibulohyoid junction. (A-J) Stills from S1 Movie. (A) All first and second pharyngeal arch muscles are present at 53 hpf. (A’) Interhyal muscles (ih) extend toward the second pharyngeal arch midline while the intermandibularis posterior muscles (imp) extend from the first arch to the anterior edge of the second arch. (B,B’) Around 55 hpf, the tips of intermandibularis posterior and interhyal muscles on each side of the head connect (arrows). (C,C’) Before 60 hpf, the left and right sides meet in the midline, connecting the four muscles of the mandibulohyoid junction (arrowhead). (C’) Hyohyal muscles (hh) extend toward the second pharyngeal arch midline while the tips of sternohyoideus muscles pass just dorsally (arrowheads). (D,D’) Around 63 hpf, the hyohyal muscles meet end-to-end in the midline (arrowhead). (E,E’) At 72 hpf, now broader muscles sit end-to-end at the mandibulohyoid and hyohyal junctions. In *cyp26b1* mutants, the timing of jaw muscle differentiation is normal (F,F’), but morphogenetic events are altered. (G,G’) Elongation toward the midline by intermandibularis posterior and interhyal muscles is slower (compare arrows to those in B’). (H,H’) The intermandibularis posterior and interhyal muscles connect poorly across the midline (distance between arrows = 46.5 μm, compare between arrows in C’ = 38.2 μm). (I,I’J,J’) Muscle fibers extend into the space bounded by the interhyal and hyohyal muscles (arrowheads). Both aberrant behaviors have phenotypic readouts at 72 hpf. (K) Where the muscles are narrowest at the junction between intermandibularis posterior and interhyal muscles (see arrows in C’,H’), the mandibulohyoid junction is significantly wider in mutants than in wild-type siblings. (L) There is also a significantly larger surface area in which the ends of intermandibularis posterior and interhyal muscles extend ectopically. All images ventral view, anterior to the left. Scale bar = 50 μm.

To characterize the genesis of muscle defects in *cyp26b1* mutant zebrafish, we used *in vivo* time-lapse imaging to track muscle morphogenesis. We used a *Tg(-0*.*5unc45b*:*mCherry); Tg(fli1*:*EGFP)y1* double transgenic line for live fluorescence imaging of muscles and neural crest, respectively. By 48 hpf, the interhyal and hyohyal muscle masses emerged on the ventrolateral surface of the second pharyngeal arch and elongated toward the midline (See S1 Movie). Around 51 hpf, two bilateral muscle masses formed on the ventrolateral surface of the first pharyngeal arch. In the next few hours, muscle fibers extended between these masses across the midline to form the intermandibularis anterior muscle. The nascent intermandibularis posterior muscle fibers elongated posteriorly from each mass toward the second pharyngeal arch (See S1 Movie). By 53 hpf, all of these first and second pharyngeal arch jaw muscles were present in both wild-type and *cyp26b1* mutant embryos (Fig 3A and 3F). Thus, Cyp26b1 is dispensable for the initial formation of these muscle masses.

As morphogenesis continued the bilateral intermandibularis posterior muscles, from the first pharyngeal arch, integrated in a central four-way junction with the interhyal muscles, of the second pharyngeal arch (S1 Movie, wild-type; Fig 3A–3C). In wild-type embryos, the intermandibularis posterior muscles, upon encountering the anterior edge of the second arch and the medial tips of the elongating interhyal muscles, ceased their rostrocaudal elongation and continued toward the midline with the interhyal muscles (Fig 3B). All four of these muscles arrived in the midline roughly at the same time, around 57 hpf. This “mandibulohyoid junction” (Fig 3C, arrowhead) was situated anteriorly and ventrally in the midline of the second pharyngeal arch, where neural crest cells surrounded the muscle tips (S2 Fig). Lastly, by about 63 hpf, the bilateral hyohyal muscles elongated ventrally past the somite-derived sternohyoideus muscles to reach each other in the midline and form the hyohyal junction (Fig 3D’, arrowhead). Together, these data showed that the attachments for eight muscles, all within the second pharyngeal arch midline, form in a period of roughly ten hours.

Loss of Cyp26b1 function had a profound effect on the connection of these muscles without altering the initial muscle dynamics. As in wild-type embryos, intermandibularis posterior muscles encountered the second arch neural crest and interhyal muscles around 55 hpf in *cyp26b1* mutants (Fig 3B and 3G). However, the elongation of interhyal muscles toward the midline was retarded by loss of Cyp26b1 function, (S1 Movie). Their medial tips were further apart at 55 hpf (compare arrows in Fig 3B’ and 3G’), and these and intermandibularis posterior muscles made no connection at the midline as late as 60 hpf. Meanwhile, intermandibularis posterior muscles continued to elongate posteriorly beyond the anterior edge of the second arch in Cyp26b1-deficient embryos (Fig 3I and 3J). Fibers could be observed extending over the neural crest cells of the medial second arch or overlapping with the medial tip of the interhyal muscle. Subsequently, muscle fibers became more disorganized rather than bundling tightly at the mandibulohyoid junction. Later muscle phenotypes indicated that some muscle fibers at the tips of interhyal muscles fan out or split off as well (see Fig 1B–1D). These results demonstrated that Cyp26b1 function is needed for the muscle cell movements necessary to integrate the ventral pharyngeal musculature, particularly at the mandibulohyoid junction, which forms the focus of this manuscript.

To quantify mandibulohyoid junction defects, we measured jaw muscles in 72 hpf embryos (Fig 3E and 3J). By this point the left and right intermandibularis posterior and interhyal muscles connected in the midline in nearly all mutants. However, the width across the mandibulohyoid junction (see arrows in Fig 3C’ and 3J’) was 12.1 μm wider in the average mutant (44.2 μm, n = 11) relative to wild-type fish (32.1 μm, n = 49, Fig 3K). The bundling of muscle fibers at the mandibulohyoid junction in wild-type embryos meant that muscles rarely overlapped or projected ectopically. In the average *cyp26b1* mutant, overlapping muscles and ectopic muscle fibers covered an area of 401.9 μm^2^ (n = 11), compared to 83.7 μm^2^ in wild-type fish (n = 49, Fig 3L). Together, these findings suggested that Cyp26b1 functions to promote the proper extension of interhyal muscles into the midline and to stop the posterior elongation of intermandibularis posterior muscles at the anterior edge of the second arch.

### Cyp26b1 functions in the neural crest to promote craniofacial musculoskeletal patterning

**Fig 4:**
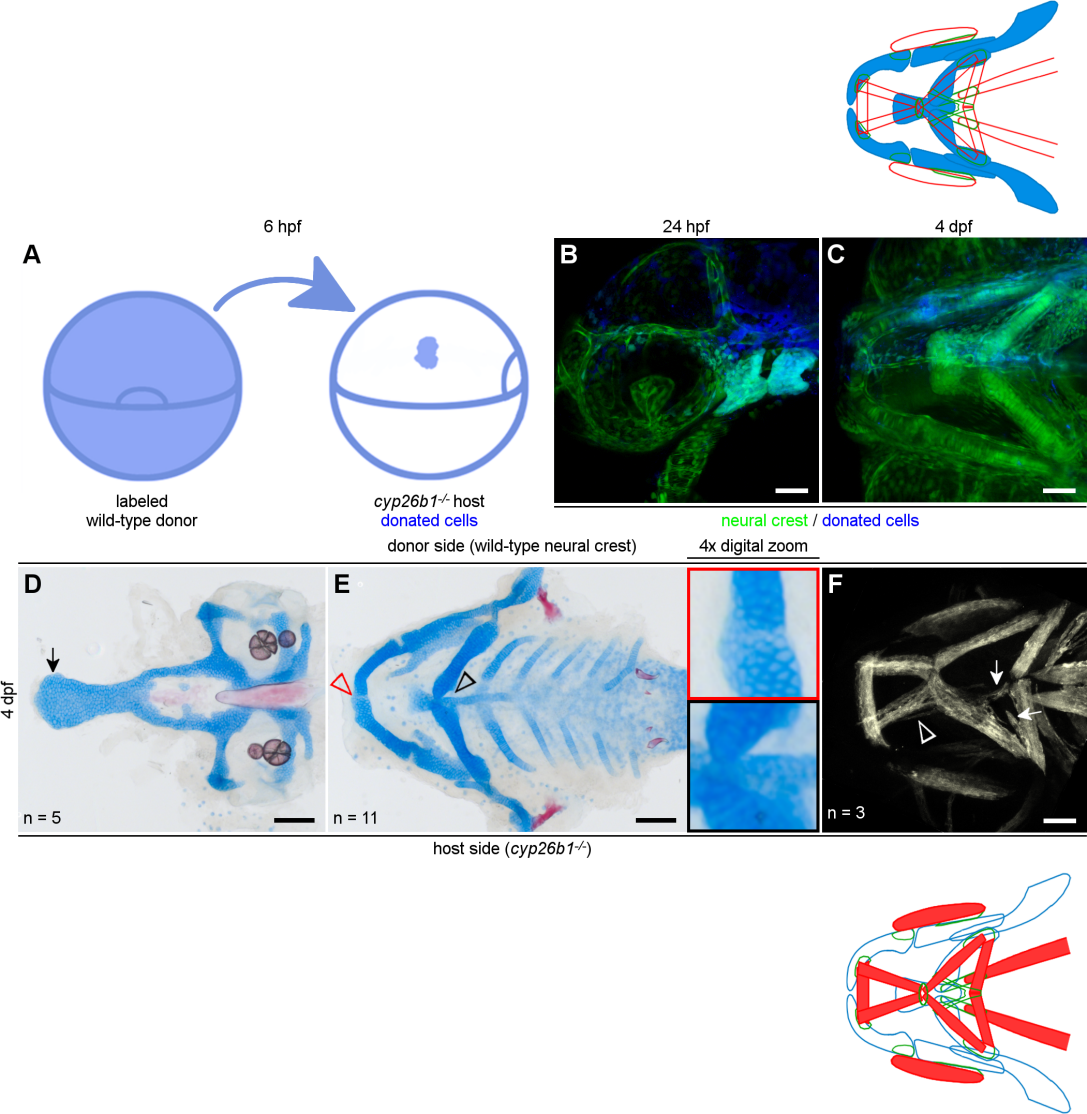
Cyp26b1 functions in the neural crest to promote craniofacial musculoskeletal patterning. (A) Schematic for transplantation of embryonic cells at 6 hpf. The blue area shown on the right side of A will contribute to the neural crest on one side of the head (B, C). (D-E) Donated neural crest cells improve skeletal phenotypes in a *cyp26b1* mutant host. We saw a wider palate with a flared ethmoid plate in mutant hosts (arrow in D). (E) Depletion of Alcian-positive cartilage matrix indicates partial rescue of Meckel’s cartilage fusion (red arrowhead/red inset) and ceratohyal morphogenesis and fusion at the midline is rescued on the donor side (black arrowhead/black inset). (F) On the side of the head without donated neural crest, muscles project ectopically (arrows) and muscle fiber bundles split (arrowhead), compared to normal phenotypes on the donor side. Lateral view in B. All other images ventral view, anterior to the left. Scale bars = 50 μm.

Because loss of Cyp26b1 disrupts skeletal and muscle morphogenesis, we performed genetic mosaic experiments to test the hypothesis that Cyp26b1 functions in neural crest cells to mediate musculoskeletal development. We transplanted cells from wild-type embryos into *cyp26b1* mutant embryos at the onset of gastrulation (Fig 4A). At 24 hours post-fertilization, we selected individuals in which there were large contributions of wild-type crest to the first- and second pharyngeal arches (Fig 4B). We subsequently evaluated skeletal and muscular phenotypes of these individuals at 4 dpf (Fig 4C–4F).

Even though donated neural crest cells contributed to one side of the head, they were able to affect midline skeletal phenotypes in each of 11 mutant hosts. Almost half of our neural crest transplants restored the flared shape of the ethmoid plate (Fig 4D, n = 5/11). Donated crest efficiently rescued the symphysis of Meckel’s (Fig 4E, red arrowhead and inset, n = 7/11) and ceratohyal (Fig 4E, black arrowhead and inset, n = 8/11) cartilages completely or partially. These results indicated that neural crest cells require Cyp26b1 function for ethmoid plate, Meckel’s cartilage, and ceratohyal development.

Consistent with our hypothesis, we found that Cyp26b1 function within the neural crest restored proper jaw muscle patterning. Though donated wild-type neural crest cells could completely rescue mutant muscle phenotypes (n = 1/6), more frequently individuals displayed unilateral improvement (Fig 4F, n = 3/6). In these embryos, jaw muscles looked wild-type on the donor side of the head. These results demonstrated that Cyp26b1 function within the neural crest integrates musculoskeletal development in the face.

### Loss of Cyp26b1 function disrupts cranial tendons

**Fig 5:**
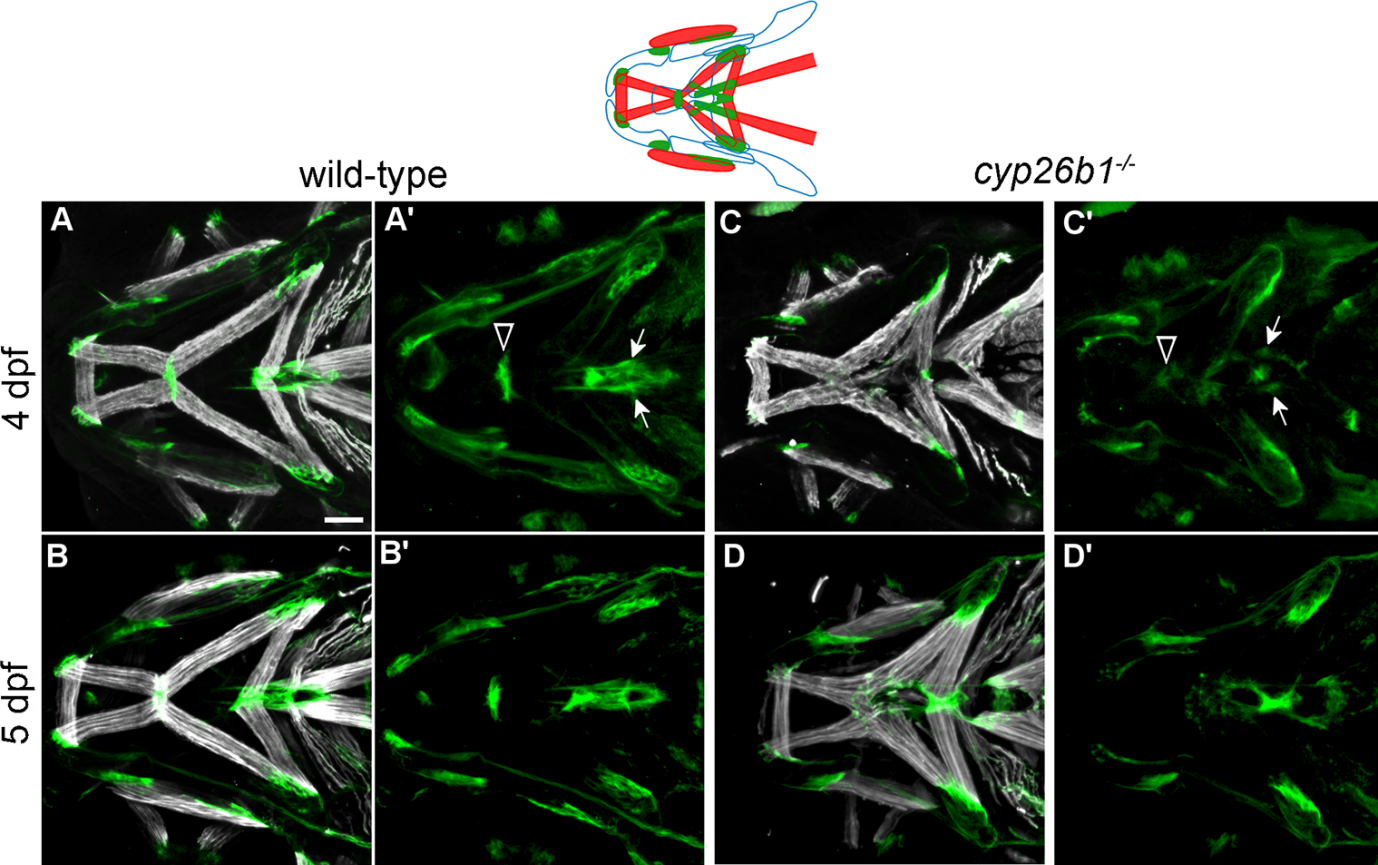
Loss of Cyp26b1 function disrupts cranial tendon differentiation. (A-B) Tsp4b (green) is enriched at the muscle attachment sites in wild-type zebrafish at 4 and 5 dpf. (C) At 4 dpf, *cyp26b1* mutants display Tsp4b at jaw muscle attachments, though weakly at the mandibulohyoid junction (arrowheads in A’,C’) and sternohyoideus tendons (arrows in A’,C’). (D) At 5 dpf, punctate deposits of Tsp4b can be seen at all ectopic points of jaw muscle attachment. All images ventral view, anterior to the left. Scale bar = 50 μm.

The mandibulohyoid junction forms within a population of *sox9a*-negative neural crest mesenchyme just ventral to the boundary between the ceratohyal cartilage condensations (S2 Fig and S3 Fig). We sought to test the hypothesis that tendons, a neural crest derivative, are defective in *cyp26b1* mutants. In wild-type embryos, differentiated Tsp4b-positive tendon tissue was greatly enriched at all cranial muscle attachment points at 4 and 5 dpf (Fig 5A and 5B). Tsp4b was enriched in *cyp26b1* mutant embryos at most of the jaw muscle attachment points at 4 dpf (Fig 5C), though the mandibulohyoid junction (arrowheads in Fig 5A and 5C) and sternohyoideus-anchoring tendons (arrows in Fig 5A and 5C) were difficult to detect. In 5-day-old mutant larvae, all visible jaw muscle attachment points, including ectopic attachments, displayed distinct Tsp4b deposition (Fig 5D). These observations suggested that loss of Cyp26b1 function disrupts the morphology of those tendons that associate with mispatterned muscles.

### Patterning of head tendon progenitors requires Cyp26b1

**Fig 6:**
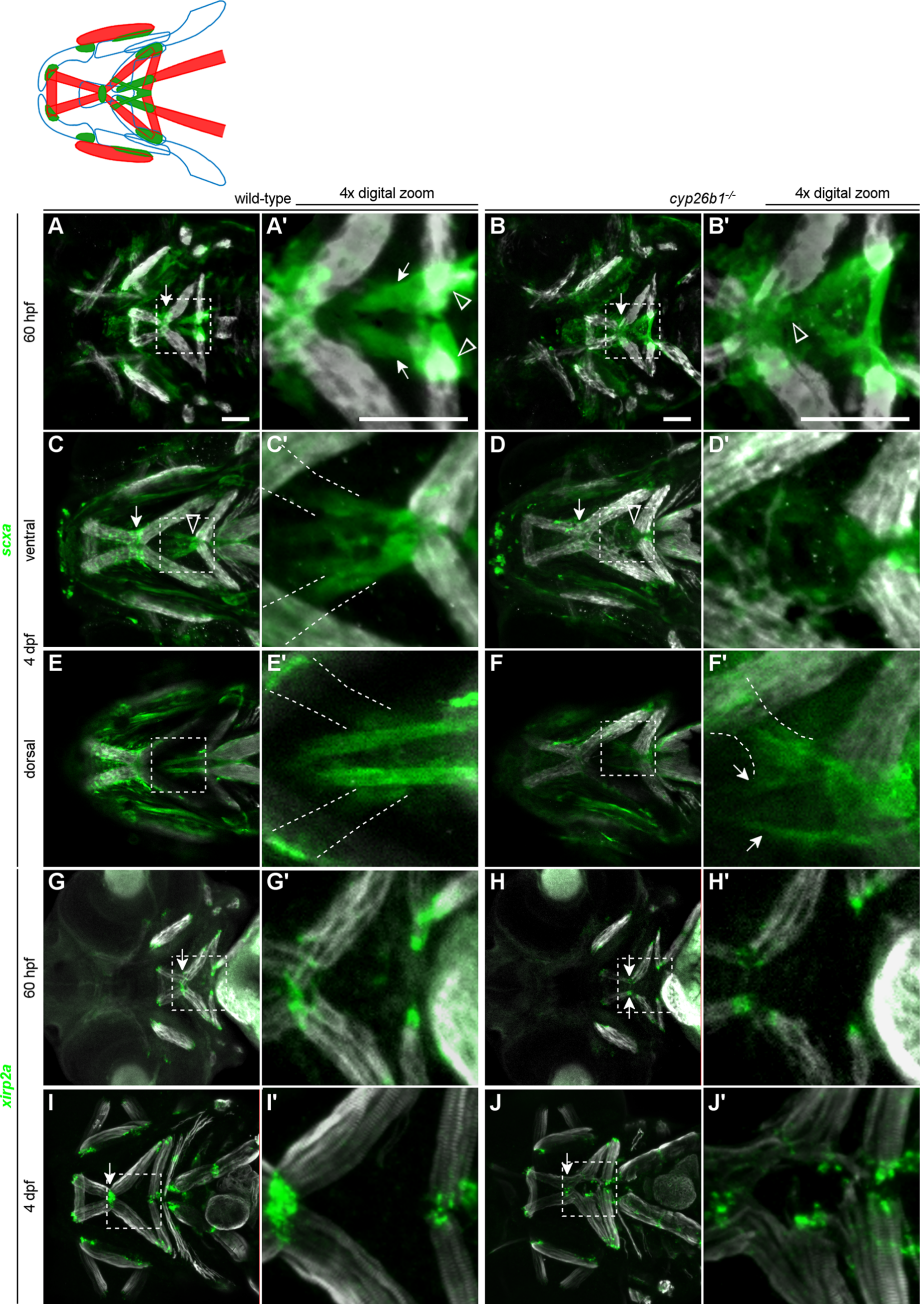
Patterning of *scxa*-expressing head tendon progenitors is disrupted in *cyp26b1* mutants. (A) At 60 hpf, bright puncta of condensing scxa-positive tenoblasts form in a line between the intermandibularis posterior and interhyal muscles (arrow), and other tenoblasts populate a mass of midline cells from the oral ectoderm to the mandibulohyoid junction. (A’) In the second pharyngeal arch, the ends of the hyohyal (arrowheads) and sternohyoideus (arrows) muscles are labeled by *scxa*-positive condensations. (B) In *cyp26b1* mutants, the pattern of *scxa* expression suggests less condensation at the mandibulohyoid junction (arrow) and less separation of anterior and posterior tenoblasts. (C) At 4 dpf, the mandibulohyoid (arrow) and hyohyal (arrowhead) junctions express *scxa* strongly, and most muscle attachment points are *scxa*-positive. Notably, two spurs extend from the hyohyal junction (C’, outlines) and insert near the anterior edge of each interhyal muscle (E’, outlines). (E,E’) The sternohyoideus tendons insert in the midline, just dorsal to the hyohyal junction structures. (D) Four-day-old mutants display some *scxa* expression at most muscle attachment points, but expression associated with the intermandibularis muscles (arrow) and the hyohyal junction (arrowhead) is reduced. (D’) The spurs of the hyohyal junction are highly dysmorphic. (F’) The sternohyoideus tendons are rod-like but hypoplastic (arrows). (G,I) Cells expressing *xirp2a* are present at the tips of all cranial muscles at 60 hpf and 4 dpf. (H,J) Cells at muscle tips are also labeled with *xirp2a* in *cyp26b1* mutants. All images ventral view, anterior to the left. Scale bars = 50 μm.

Alterations to Tsp4b deposition in 3-day-old *cyp26b1* mutant embryos led us to question whether tenoblasts are disturbed during the period of jaw muscle morphogenesis. Using a *fli1*:*EGFP;scxa*:*mCherry* double transgenic line, we observed that *scxa-*positive neural crest cells were condensing along the forming mandibulohyoid junction at 60 hpf (Fig 6A, arrow). Four other *scxa*-positive condensations resided more posteriorly in the medial second pharyngeal arch, two slightly dorsal sternohyoideus tendons which attach to the ceratohyal cartilage at a region of *scxa*:*mCherry;sox9a*:*EGFP* double positive cells (arrows in Fig 6A’, S3 Fig) and two smaller condensations labeling the medial tips of the hyohyal muscles (arrowheads in Fig 6A’). An elongated group of *fli1:EGFP* expressing cells defined the insertion of the sternohyoideus muscles (S4A–S4D Fig). These brightly *fli1*:*EGFP*-positive cells separated the two ceratohyal cartilage condensations, and also separated anterior, ventral tenoblasts from the posterior, dorsal sternohyoideus tendon condensations (arrowheads in S4B and S4C Fig). At 4 dpf, *scxa*:*mCherry* expression labeled all cranial tendons and ligaments (Fig 6C). The mandibulohyoid junction (arrow in Fig 6C) and a small population of *scxa-*positive mesenchyme on the periphery of the intermandibularis posterior muscles displayed the *scxa* reporter brightly. Expression of the *scxa* reporter was also strong at the hyohyal junction (Fig 6C’, arrowhead in Fig 6C), and two *scxa*-positive spurs extended from between the hyohyal muscles (outlines in Fig 6C’) to the anterior edge of each interhyal muscle (outlines in Fig 6E’). Notably, these *scxa*-positive spurs displayed little to no Tsp4b deposition in wild-type embryos, though Tsp4b was enriched on every other cranial tendon and ligament (compare Fig 5B and Fig 6C). Just dorsal to the hyohyal junction structures were the long, rod-shaped sternohyoideus tendons (Fig 6E). Our findings suggested that tendon components of the mandibulohyoid junction develop from a population of anterior tenoblasts that is segregated, by at least 60 hpf, from those posterior tenoblasts that form the hyohyal junction and the sternohyoideus tendons.

In *cyp26b1* mutants, the population of *scxa*-positive neural crest cells was less organized. In mutants we saw there was less clear separation of anterior and posterior tenoblasts in the second pharyngeal arch (Fig 6B, arrowhead in Fig 6B’). Though visually it appeared that the population of tenoblasts could be expanded in mutants, cell counts indicated that 60 hpf mutants (148.67 tenoblasts, SD = 2.89, n = 3) did not have significantly more or fewer tenoblasts than siblings (159.67 tenoblasts, SD = 16.4, n = 6). Instead, the *scxa-*positive cells in 60 hpf *cyp26b1* mutants were not concentrated at the mandibulohyoid junction, as they were in wild-type embryos (arrow in Fig 6B), though condensations still formed at the sternohyoideus and hyohyal muscle tips. The structure we saw separating the ceratohyal cartilage condensations was also missing in mutants (S4E–S4H Fig). We observed bright, *fli1:EGFP* expressing cells amidst the tenoblasts, but they formed a round shape ventral to the ceratohyals instead of elongating between them (green arrowhead in S4F Fig). Four-day-old *cyp26b1* mutants did not maintain strong *scxa* expression at the mandibulohyoid and hyohyal junctions (arrow and arrowhead in Fig 6D, respectively). In fact, mutants lacked defined spurs at the hyohyal junction. Instead, a poorly organized population of *scxa-*positive cells stretched between the tips of the hyohyal muscles and the adjacent interhyal muscles, as well as ectopic muscle projections from these (detail in Fig 6D’). Four day old mutants had developed sternohyoideus tendons, but these were hypoplastic and slightly curved (arrows in Fig 6F’).

We used *in situ* hybridization to visualize expression of an early muscle-proximal tendon marker, *xirp2a*, which labels all muscle attachments [2] to further characterize tendons in *cyp26b1* mutants. At both 60 hpf and 4 dpf, *xirp2a* labeled cells at the tips of cranial muscle fibers (Fig 6G and 6I). This remained true in *cyp26b1* mutant embryos, in which *xirp2a* labeled muscle fiber tips even at ectopic points of attachment (Fig 6H and 6J). Notably, in 60 hpf mutants there was no expression of *xirp2a* in the midline where the mandibulohyoid junction should be forming. Together, these findings suggested that the *cyp26b1* mutant muscle phenotype is coincident with a defective pattern of *scxa-*positive tenoblasts, but mutants showed tendon matrix deposition over time because muscles and *xirp2a-*positive cells still promote tendon differentiation.

### Loss of Cyp26b1 function perturbs the morphogenesis of *scxa*-positive tendon progenitors

**Fig 7:**
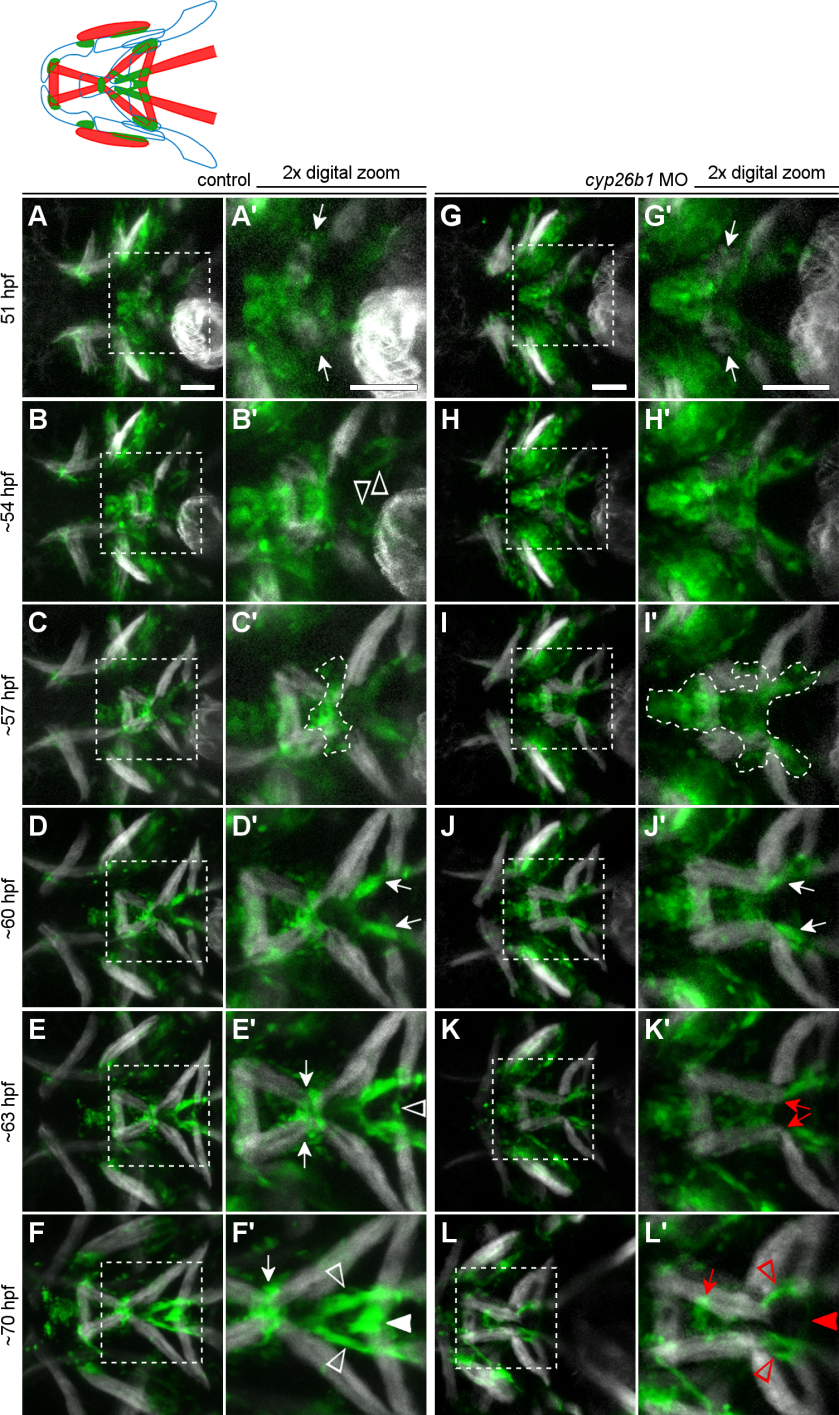
Loss of Cyp26b1 function disrupts tendon morphogenesis in the second pharyngeal arch midline. (A-L) Stills from S2 Movie. (A,A’) The emerging intermandibularis muscles (arrows) are surrounded by tenoblasts at 51 hpf (B,B’). As tenoblasts converge on the midline, intermandibularis muscles point their posterior tips medially (also see S6B Fig). Meanwhile, tenoblast masses (arrowheads) associated with the hyohyal and sternohyoideus muscles on each side have become visible. (C,C’) By 57 hpf, condensing cells are visible at the mandibulohyoid junction (outline), where *scxa-*positive cells have segregated from the more anterior group. (D,D’) By 60 hpf, condensation of the sternohyoideus tendons is apparent (arrows). (E,E’) By 63 hpf, tenoblasts attached to each hyohyal muscle connect in the midline and a condensation forms (arrowhead). (F,F’) Approaching 3 dpf, tenoblasts at the mandibulohyoid (arrow) and hyohyal (white arrowhead) junctions are highly condensed between the muscle tips, and sternohyoideus tendons are elongated (open arrowheads). (G,G”) In morpholino-injected embryos, intermandibularis muscles (arrows) emerge normally among a field of tenoblasts around 51 hpf. (H,H’) Tenoblasts migrate slowly toward the midline, and intermandibularis posterior muscles point posteriorly at 54 hpf (also see S6D Fig). (I,I’) Tenoblasts at the mandibulohyoid junction are neither condensing nor separating from adjacent tenoblasts at 57 hpf (outline). (J,J’) Despite surrounding abnormalities, sternohyoideus tendons initiate condensation around 60 hpf (arrows). (K,K’) Connections between intermandibularis posterior muscles and sternohyoideus tendon condensations persist, and at 63 hpf the muscle tips (red arrows) now extend past mandibulohyoid tenoblasts. (L) Compared to controls, the sternohyoideus tendons are poorly condensed and elongated by 70 hpf, and both intermandibularis posterior and both hyohyal muscles connect to either end of these tendon rudiments. (L’) Mesenchymal tenoblasts sit between the intermandibularis posterior muscles (arrow) and no condensation has formed connecting the hyohyal muscles (red arrowhead). All images ventral view, anterior to the left. Scale bar = 50 μm.

Based on the disruption of mature tendon morphology in *cyp26b1* mutants, we hypothesized that loss of Cyp26b1 function disrupts tenoblast behaviors. Live imaging in *Tg(-0*.*5unc45b*:*EGFP);scxa*:*mCherry* double transgenic control embryos revealed dynamic populations of tenoblasts in the ventral midline of the jaw (S2 Movie, Fig 7A–7F). In control embryos, tenoblasts surrounded muscle fibers of the intermandibularis muscles as they formed near the midline of the first pharyngeal arch around 51 hpf (arrows in Fig 7A’, see S5A Fig for an enlarged image). Tenoblasts appear to migrate toward the midline as the intermandibularis posterior muscles elongated (Fig 7B), populating the midline prior to the muscles masses (S5B Fig, arrowhead). Coincidently, the intermandibularis posterior muscles angled toward the midline by 54 hpf, almost perpendicular to the intermandibularis muscle masses at 51 hpf (compare S6A and S6B Fig). By this time, two other tenoblast masses at the tips of the hyohyal and sternohyoideus muscles became visible (arrowheads in Fig 7B’). By 57 hpf, a bright condensation of tenoblasts localized at the anterior tip of the second pharyngeal arch (outline in Fig 7C’). Over the next few hours, this rudimentary mandibulohyoid junction took shape as tenoblasts continued to condense in the midline, and the intermandibularis posterior and interhyal muscles elongated toward the condensation. Around 60 hpf, two more bright condensations formed from tenoblasts extending from the tips of the sternohyoideus muscles (arrows in Fig 7D’). One final condensation, connecting the hyohyal muscles, formed after tenoblasts extended from each muscle tip to make contact in the midline around 63 hpf (arrowhead in Fig 7E’). By 70 hpf, the sternohyoideus tendons and tendon elements of the mandibulohyoid and hyohyal junctions appeared bright and compact (Fig 7F). Tenoblast condensation thus precedes the morphogenesis of the mandibulohyoid and hyohyal junctions.

In *cyp26b1* morpholino-injected embryos, clear tenoblast defects presaged the ectopic elongation of intermandibularis posterior muscles (S2 Movie, Fig 7G–7L). Loss of Cyp26b1 function did not appear to interfere with tenoblast specification, as tenoblasts surrounded the newly forming intermandibularis muscle fibers at 51 hpf (arrows in Fig 7G’). However, these tenoblasts failed to condense at the midline (Fig 7H–7L). At 54 hpf, the intermandibularis posterior muscles were not oriented toward the midline but rather toward the posterior or even slightly laterally (arrows in S6D Fig). Over time, muscle angle did turn toward the midline, but no tenoblast condensation was apparent at the mandibulohyoid junction at 57 hpf (Fig 7I). Instead, a contiguous, diffuse mass of tenoblasts stretched from the oral ectoderm to the sternohyoideus muscles (outline in Fig 7I’). Condensation of the sternohyoideus tendons initiated normally around 60 hpf (arrows in Fig 7J’), and the intermandibularis posterior muscles extended beyond the prospective mandibulohyoid junction to abut these condensations. By 63 hpf, the sternohyoideus tendon condensations were the only tenoblasts touching the tips of the intermandibularis posterior muscles (arrows in Fig 7K’). Loss of Cyp26b1 inhibited tenoblast condensation between the hyohyal muscles, and blocked their connection even out to 3 dpf (arrowhead in Fig 7L’). No tendon condensation was visible between the tips of the intermandibularis posterior and interhyal muscles, but mesenchymal tenoblasts sat in the midline between the intermandibularis posterior muscles (arrow in Fig 7L’). These results indicated that Cyp26b1 function is required for the precise morphogenesis of several cartilage, tendon, and muscle elements that all occupy the ventral midline of the second pharyngeal arch.

### Cyp26b1 function before 60 hpf is necessary and sufficient for mandibulohyoid junction morphogenesis

**Fig 8:**
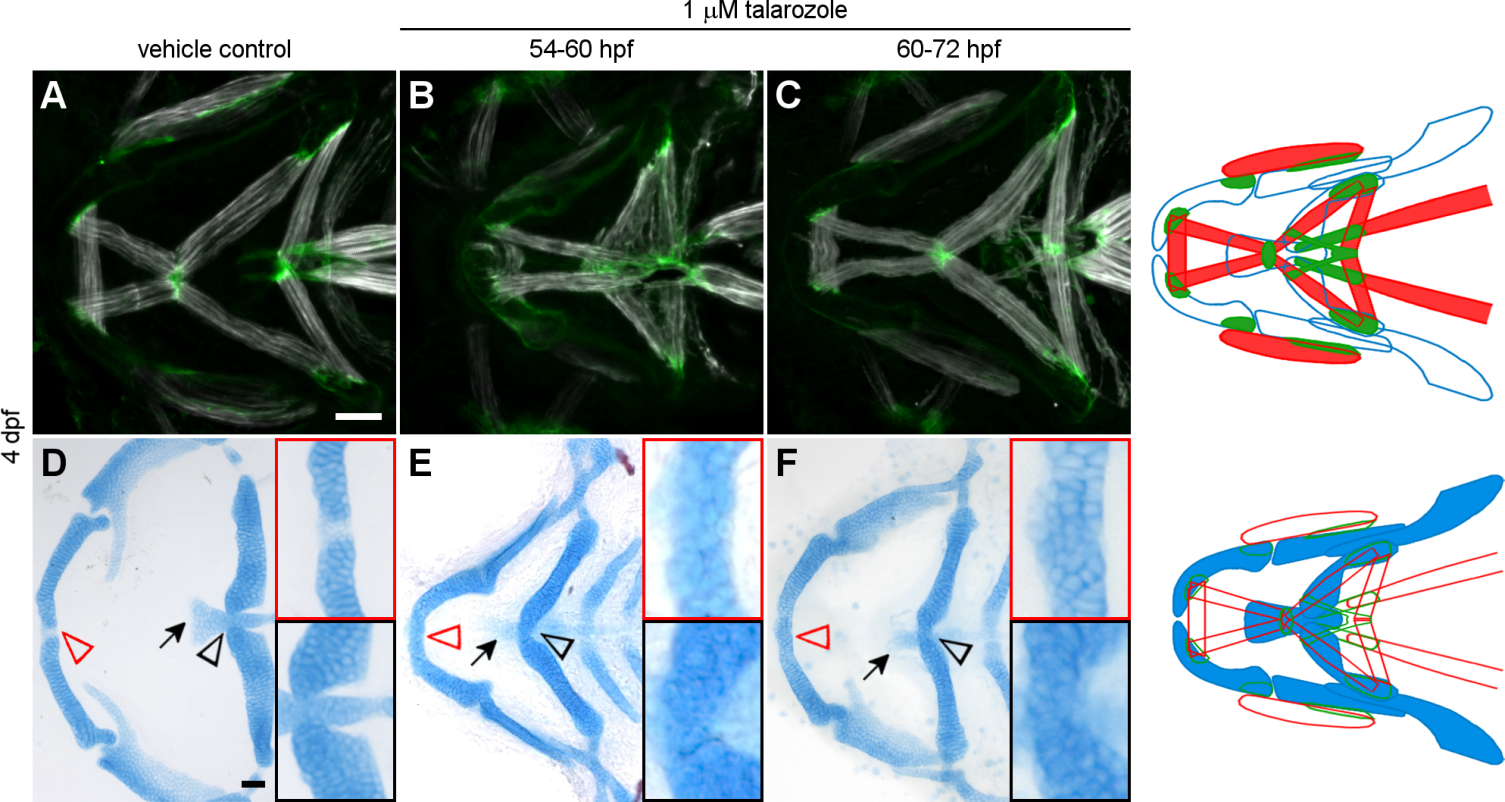
Cyp26b1 function before 60 hpf is necessary for mandibulohyoid junction formation. (A,D) Embryos raised in media containing 0.01% DMSO develop normally. (B) 100% of embryos treated with talarozole between 54 and 60 hpf displayed ectopic muscle projection in the second pharyngeal arch, and the intermandibularis posterior muscles overextended in 58.3% of embryos (n = 64). (C) Among embryos treated from 60 to 72 hpf, 76.9% had mild ectopic muscle projections, but 96.2% developed normal tendon and end-to-end muscle connections at the mandibulohyoid junction (n = 26). (E,F) Talarozole-treated embryos recapitulated *cyp26b1* mutant skeletal phenotypes of Meckel’s cartilage fusion (red arrowheads and insets), ceratohyal fusion (black arrowheads and insets), and basihyal reduction (arrows). All images ventral view, anterior to the left. Scale bar = 50 μm.

Because jaw muscle morphogenesis occurs over several hours, we sought to ascertain when Cyp26b1 function is required for this process. We treated embryos with media containing talarozole, which inhibits Cyp26b1 and its orthologs Cyp26a1 and Cyp26c1, then assessed tendon and muscle morphology at 4 dpf. Compared to controls (Fig 8A), embryos treated with talarozole during the period of tenoblast migration to and condensation at the mandibulohyoid junction (54–60 hpf) had severe phenotypes (Fig 8B). Of 64 individuals, 100% displayed ectopic muscle projections across the second pharyngeal arch, including 58.3% in which the intermandibularis posterior muscles extended past the location of the mandibulohyoid junction. The sternohyoideus muscle was examined in a subset of these, and in 51% of embryos there were fibers extending across the midline in these muscles (n = 27/53). These defects were typically more severe than those observed in *cyp26b1* mutants, but were still restricted to the ventral midline (the dorsal adductor mandibulae muscle was normal in 54/55 embryos examined, with a single stray muscle fiber in 1 fish). Overall our results suggest partial redundancy across Cyp26 enzymes in patterning the ventral musculoskeletal attachments.

Treatment during the period of sternohyoideus tendon condensation and hyohyal junction formation (60–72 hpf) affected muscle morphology weakly (Fig 8C). Of 26 individuals, 76.9% displayed mild ectopic muscle projections from the interhyal or hyohyal muscles, but 25 of 26 developed a normal mandibulohyoid junction. These data show that for proper musculoskeletal patterning, Cyp26b1 function is required when mandibulohyoid tenoblasts are migrating and condensing.

We also evaluated the effects of Cyp26 inhibition on the cranial skeleton at 4 dpf. Control larvae had normal phenotypes (Fig 8D). Talarozole treatment from 54–60 hpf recapitulated much of the jaw skeletal phenotype of *cyp26b1* mutants. These embryos consistently displayed fused Meckel’s (Fig 8E, red arrowhead and inset) and ceratohyal cartilages (Fig 8E, black arrowhead and inset), and loss or severe hypoplasia of the basihyal cartilage (Fig 8E, arrow). Embryos treated from 60–72 hpf also had midline cartilage fusions and basihyal cartilage hypoplasia (Fig 8F). These findings suggested that Cyp26b1 continues to function in cartilage development after the mandibulohyoid junction forms, and that the mandibulohyoid junction can form normally despite deformity of the basihyal and ceratohyal cartilages.

### Cyp26b1 expression separates anterior and posterior muscle attachments in the midline of the second pharyngeal arch

**Fig 9:**
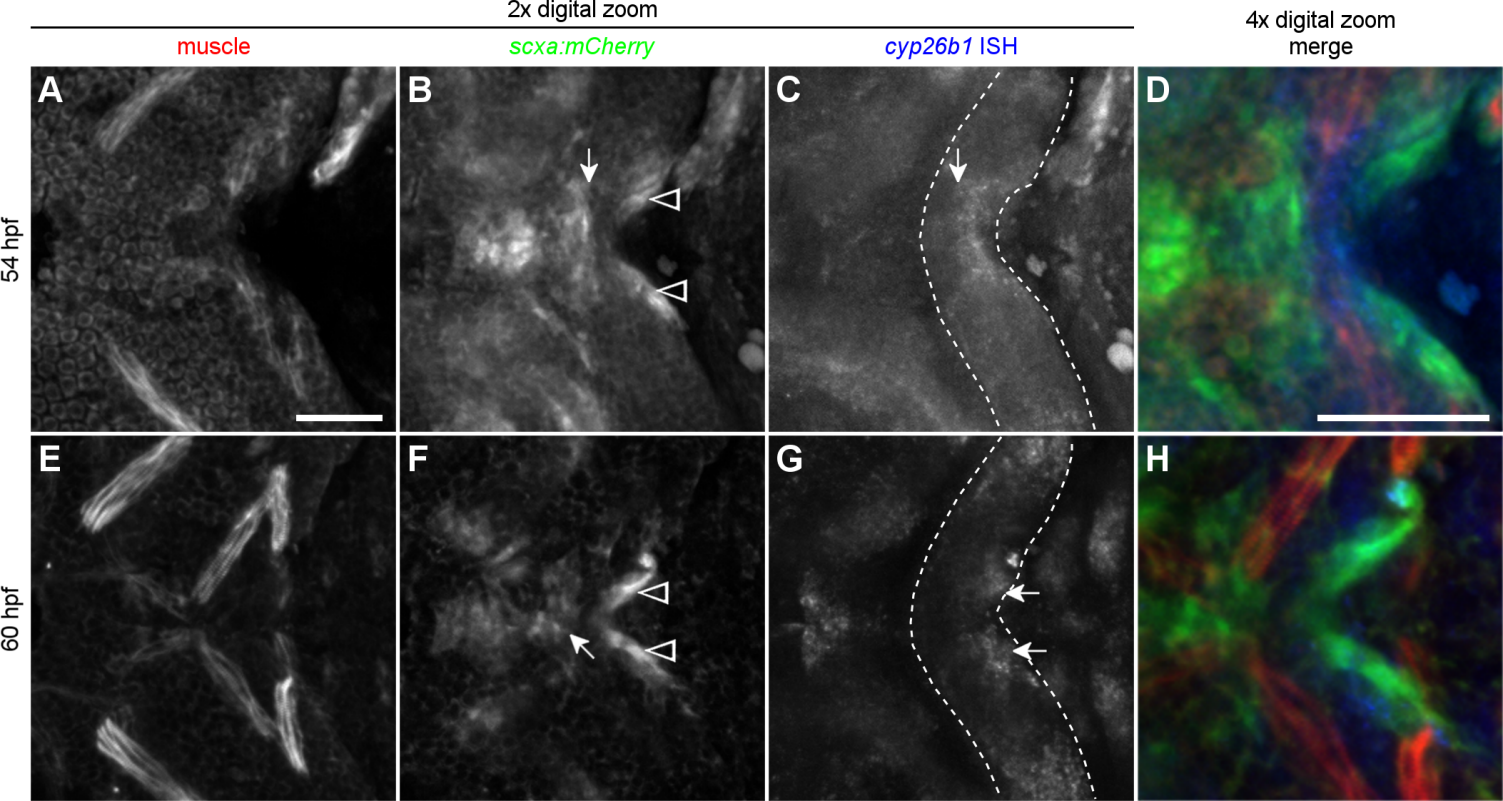
Cells expressing *cyp26b1* separate anterior and posterior tenoblast masses between 54 and 60 hpf. (A-D) 54 hpf zebrafish embryo. (B) Tenoblasts of the future mandibulohyoid junction (arrow) display a flattened posterior boundary and maintain a small distance from tenoblasts lining the posterior edge of the second pharyngeal arch (arrowheads). (C) With the shape of the second arch visible (outline) one can see that cells expressing *cyp26b1* line the posterior edge of the arch midline and display a flattened anterior boundary abutting the mandibulohyoid tenoblasts (arrow). (D) With channels combined, *cyp26b1* expression (blue, *in situ* hybridization) labels non-tenoblast cells separating tenoblasts (green, anti-mCherry antibody in *scxa*:*mCherry* transgenic) and second arch muscles (red, MF20) that are mostly or entirely c*yp26b1*-negative. (E-H) 60 hpf zebrafish embryo. (F) Tenoblasts are more compact at the forming mandibulohyoid junction (arrow), and further separated from the sternohyoideus tendon condensations (arrowhead). (G) Strong *cyp26b1* expression now labels bilateral cell masses of the posterior edge of the second arch (arrows). (H) Non-tenoblast cells expressing *cyp26b1* sit anterior to the sternohyoideus tendon condensations on each side, facing the nearby interhyal muscles. All images ventral view, anterior to the left. Scale bar = 50 μm.

Cyp26b1 function is required in the neural crest during the process of tenoblast migration and condensation. We turned to *in situ* hybridization to determine if there were subpopulations of neural crest cells expressing *cyp26b1* at these times. Intriguingly, at 54 hpf, a band of *cyp26b1*-positive cells filled the midline of the posterior second arch between the posterior edge of the mandibulohyoid tenoblast mass (Fig 9B, arrow) and the sternohyoid tenoblast masses (Fig 9B, arrowheads). There were few, if any, *cyp26b1*-expressing tenoblasts. The mandibulohyoid tenoblasts appeared entirely *cyp26b1*-negative. At 60 hpf, the gap between condensing mandibulohyoid tenoblasts (Fig 9F, arrow) and sternohyoid tenoblasts (Fig 9F, arrowheads) had grown. Strong *cyp26b1* expression labeled bilateral cell masses in the posterior second arch (Fig 9G, arrows). Again, there appeared to be little overlap between the *scxa* transgenic and *cyp26b1* expression. This pattern of expression, with *cyp26b1* adjacent to *scxa*:*mCherry* expressing cells, is conserved at the tips of other muscles which are not disrupted in *cyp26b1* mutants (S7 Fig). Thus, *cyp26b1* may be a general marker of mesenchyme adjacent to developing tendon, but does not appear to be required at all of these sites. Together, our results indicated that Cyp26b1 functions in a mesenchymal population of neural crest cells to promote separation and condensation of tendons in the ventral midline of the second pharyngeal arch.

This expression pattern suggests that *cyp26b1* is required in a neural crest population outside of tenoblasts for their condensation. If this were the case then wild-type tenoblasts should fail to condense appropriately in an otherwise mutant environment. We performed transplants using small numbers of cells (10–20) from *fli1*:*EGFP;scxa*:*mCherry* hosts into embryos generated from matings of *cyp26b1* carriers. In rare instances (n = 3), we found that the ventral midline of the second arch was only populated by *fli1*:*EGFP;scxa*:*mCherry* double-positive tenoblasts (S8 Fig, insets, asterisks mark the oral opening). When these cells were transplanted into wild-type embryos (n = 2), the tenoblasts associated tightly with the muscles and elongated at the mandibulohyoid junction (S8A Fig, arrowhead). In the instance when these wild-type cells were transplanted into a mutant host, most tenoblasts were localized in a region medial to the intermandibularis posterior muscles (S8B Fig, arrow). This region precisely matches the region where tenoblasts are ectopically localized in *cyp26b1* mutants (See Fig 7). Additionally, tenoblasts at or near where the mandibulohyoid junction would form are not elongated (S8B Fig, arrowhead). Collectively, our findings support the model in which *cyp26b1* is required neural crest autonomously but tenoblast non-autonomously for tendon condensation. Tools to specifically target this previously undescribed *cyp26b1*-expressing mesenchyme do not currently exist and will be essential to directly determine the autonomy of the mutation.

## Discussion

Here we provide *in vivo* characterization of developing musculoskeletal attachments in a living embryo and demonstrate the involvement of retinoic acid in this process. We found that Cyp26b1 function is essential for ventral cranial muscle patterning, but not specification. Genetic mosaic analysis demonstrated that neural crest cells required Cyp26b1 function for proper muscle patterning. Time-lapse confocal imaging showed that neural crest derived tenoblasts appear to migrate to and condense in regions that predict muscle attachments. In *cyp26b1* mutants, tenoblasts are generated, yet fail to condense appropriately at those muscle attachments that are disrupted. Furthermore, crucial events of tendon and muscle morphogenesis appear to be orchestrated by non-tenoblast neural crest cells expressing *cyp26b1*.

### Cranial muscle and tendon morphogenesis are concerted processes that require Cyp26b1 function

Our work is consistent with that of others showing that neural crest cells have critical influences on muscle morphology in the face. Transplanted neural crest cells confer their axial [4] and species-specific [5] identities upon cranial muscles. Our findings provide strong evidence for region specific patterning of tendons, with the ventral midline, particularly at the junction of the 1^st^ and 2^nd^ arch, requiring Cyp26 function. What other region specific cues may be present that pattern craniofacial tendon development are currently unknown. However, loss of Edn1 signaling has been shown to dramatically disrupt ventral musculature and skeletal elements [16]. While tendons were not analyzed in this study, it is highly likely that neural-crest-derived tenoblasts use the same patterning information. Edn1 and Bmp act in opposition to Jagged-Notch signaling to pattern the ventral to dorsal axis within the skeletogenic neural crest [17]. It will be of great interest to determine the tendon phenotypes in mutants within these pathways.

In mouse and zebrafish, and presumably all vertebrates, neural crest gives rise to Scx-positive cranial tendons and ligaments [2, 3], unlike other regions of the body where tendon, bone and muscle all derive from mesoderm populations [18, 19]. Our work with that of others demonstrates that, regardless of the source of the progenitors, tendons are critical for muscular patterning. In the chick, ablation of tendon primordia results in mispatterning of the limb musculature [6]. We find in our *cyp26b1* mutants similar overextension of specific muscles associated with highly disrupted tendons. Our work demonstrates that the presence of tenoblasts is not sufficient for muscular patterning and that condensation appears to be a critical step in musculoskeletal integration.

In no system are the cell dynamics underlying musculoskeletal integration understood. Our time-lapse imaging suggests that tendon progenitors migrate to muscle attachment points closely followed by the muscle fibers themselves. Because musculoskeletal patterning in the head is neural crest dependent, this behavior suggests that muscle patterning by neural crest [4, 5] could be due to short range cues from tenoblasts. Tendon progenitors form in *cyp26b1* mutants but fail to condense appropriately, particularly at the mandibulohyoid junction. Muscle fibers, still in close contact with tenoblasts, fail to elongate toward the midline appropriately without tenoblast condensation. These results could be explained by one of two models. In the first model, the tenoblasts themselves regulate the elongation of muscles, with tenoblast condensation depending upon *cyp26b1*-expressing cells. In the second model, muscle elongation is independent of tenoblasts, with tenoblast and muscle cell behaviors both being directly or indirectly influenced by *cyp26b1*-expressing cells. Data from the chick limb would favor the first model, but experiments in which tenoblasts are specifically deleted will be required to answer this question in the head. Given the dearth of knowledge of this process, the *cyp26b1* mutant is likely to provide key insights into musculoskeletal integration.

### Retinoic acid regulates musculoskeletal integration of the second pharyngeal arch

Our results imply a specific role for Cyp26b1 in mandibulohyoid junction formation that is not autonomous to tenoblasts or muscles. Expression of retinoic acid pathway components is highly dynamic, and for much of early pharyngeal arch development *cyp26b1* expression is broad [13]. However, at 54–60 hpf, when Cyp26b1 function is required for tenoblast condensation, c*yp26b1* expression in the second pharyngeal arch is restricted predominantly to non-tenogenic midline neural crest cells. This differs greatly from RA dynamics reported in chick limbs, where a Cyp26 enzyme is expressed in tendons themselves at the interface with muscle and RA promotes apoptosis of ectopic muscle fibers between adjacent tendons [20]. It remains to be determined if this is a general difference between the role of RA signaling in musculoskeletal development of the head versus the limb.

Our work strongly suggests that a novel population of neural crest modulates musculoskeletal integration in the second pharyngeal arch. We have found that neural crest cells expressing *cyp26b1* occupy a region that excludes *scxa*-positive tenoblasts. Cyp26b1 function in this region of exclusion likely promotes tendon condensation anteriorly and posteriorly. In live imaging of *cyp26b1*-depleted embryos, tenoblasts are spread widely around the mandibulohyoid junction and across this exclusion region. The intermandibularis posterior and interhyal muscles elongate and connect within this field, but neither these muscles nor their connection drives the tenoblasts to condense. Instead, mandibulohyoid tenoblasts remain a loose mesenchymal population, and the tips of the intermandibularis posterior and interhyal muscles migrate toward the sternohyoideus tendon condensations. Furthermore, the sternohyoideus tendons fail to condense at the same rate as in controls despite contact with supernumerary muscle tips. Thus, *cyp26b1* mutants have cranial muscles that maintain tenoblast populations, but these muscles are not sufficient for tendon differentiation in the absence of the *cyp26b1-*expressing cell population we describe. We propose that signals from these neural crest cells are critical for promoting specific sites of tendon condensations, which then organize the musculature.

#### The role of retinoic acid in tendon development

How might excess RA inhibit cranial tendon condensation? Much work is needed to answer this question, partly because the function of Cyp26b1 is likely to range beyond the cells in which it is expressed. The changes in local RA signaling resulting from loss of Cyp26b1 function could alter adjacent populations of neural crest cells and tenoblasts, and directly or indirectly affect tenoblast condensation.

RA most often functions by causing retinoic acid receptor binding to DNA response elements where they regulate gene transcription. In mice, dual-positive *Sox9;Scx* cells form bony eminences that connect limb tendons to the bone proper [21, 22]. Tenoblast conditional knockout of *Sox9* via *Scx*:*Cre* results in a loss of bone eminences, but not a loss of tendon [21, 22], demonstrating a requirement for *Sox9* in proper tendon-to-bone attachment. We do not find *scxa;sox9a* double positive cells at the mandibulohyoid junction making this an unlikely cause of these dramatic muscle attachment defects. However the attachment site for the sternohyoideus tendon is comprised of double positive cells. Therefore, the defects in mandibulohyoid and sternohyoideus attachments could be mechanistically distinct. A more detailed characterization of this *scxa;sox9a* double positive lineage will be necessary to determine its potential role in musculoskeletal integration in the zebrafish face.

Our *cyp26b1* mutants initiate *scxa* expression normally and the specification of muscle appears to follow a normal developmental trajectory. This suggests that mutants do not suffer a general musculoskeletal developmental delay but, rather, that RA modulates the differentiation of tendons downstream of initial *scxa* expression. Muscle contractions maintain Fgf and Tgfβ signaling necessary for maturation of tendon tissue [10]. It will be of great interest to explore whether RA signaling modulates Fgf or Tgfβ signaling in the zebrafish head or if RA directly regulates Fgf and Tgfβ target genes in tenoblasts.

Additionally, RA has also been shown to directly promote neurite outgrowth [23] and act as a neurite chemoattractant, independent of its transcriptional regulatory mechanisms [24]. This suggests an alternative model in which focally elevated RA directly disrupts cell movements required for condensation. While future research is required to distinguish these possibilities, inhibiting Cyp26 function precisely during the 6-hour time window when the mandibulohyoid junction is condensing disrupts this morphogenesis, arguing for more direct regulation by RA.

The ability to, at least partly, disentangle the effects of Cyp26 on muscle versus skeletal morphogenesis is surprising. We hypothesized that abnormal formation of the mandibulohyoid junction subsequently limited muscle contractile forces necessary for joint formation [25] and skeletal morphogenesis [26–29]. The basihyal and ceratohyal cartilages form immediately adjacent to the mandibulohyoid junction. Our hypothesis suggested that the reduction of the basihyal cartilage and the midline fusion of the ceratohyal cartilage in *cyp26b1* mutants could both be explained by the muscle phenotype. However, blocking Cyp26 function from 60–72 hpf recapitulates the skeletal phenotypes while leaving the mandibulohyoid junction largely intact. We cannot exclude the possibility that the mandibulohyoid junction is necessary for proper joint and basihyal morphogenesis, but our results strongly suggest that Cyp26b1 has a separate function to promote joint and cartilage fates in the midline.

Our work also combines with other Cyp26b1 loss-of-function models to suggest an evolutionarily conserved RA signaling mechanism for patterning facial muscle. In mouse, palate and tongue development requires *Cyp26b1* [30]. Loss of Cyp26b1 function increases RA signals to tongue muscles in the ventral midline and activates RA-responsive gene expression ectopically in neural crest cells surrounding these muscles. Besides the glossal muscle, all of the dysmorphic muscles are part of the anterior suprahyoid. Like the affected muscles in zebrafish *cyp26b1* mutants, they form a complex of first- and second-arch branchiomeric musculature that attaches to the anterior hyoid skeleton. The diverse morphologies of these mouse and fish muscles belie their similar origins and likely molecular conservation. Differential expression of *Fgf10* in the tongues of *Cyp26b1* mutant mouse embryos suggests specific Fgf signaling components that could be investigated in zebrafish *cyp26b1* mutants.

Collectively, our work provides novel inroads regarding the genetic underpinnings of craniofacial muscle patterning by neural crest cells. Furthermore, we detailed the dynamic morphogenesis of craniofacial muscles and tendons. Cyp26b1 promotes the morphogenesis of apparently several different neural crest derived structures in the second pharyngeal arch that serve to organize the muscles responsible for the operation of the zebrafish lower jaw. Our findings are illustrative of the interactivity and fine patterning of tissues in the vertebrate head.

## Methods

### Ethics statement

Zebrafish stocks were maintained and embryos were raised according to established protocols [31] with approval from the University of Texas at Austin Institutional Animal Care and Use Committee. The approved protocol includes authorization for embryonic zebrafish euthanasia by overdose with MS-222/Tricaine.

### *Danio rerio* (zebrafish) care and husbandry

The *cyp26b1*^*b1024*^ allele was recovered from a forward genetic screen at the University of Oregon. The *Tg(fli1*:*EGFP)y1* [32], *Tg(-0*.*5unc45b*:*EGFP)* [33] and *Tg(foxP2-enhancerA*:*EGFP)* [34] transgenic lines are referred to as *fli1*:*EGFP*, *503unc*:*EGFP* and *sox9a*:*EGFP*, respectively, throughout the text. The *503unc* promoter fragment [33] and Tol2Kit materials and protocols [35] were used to construct the *Tg(-0*.*5unc45b*:*mCherry)* transgenic line, referred to as *503unc*:*mCherry*. The *Tg(scxa*:*mCherry)* line is referred to as *scxa*:*mCherry* throughout the text. For pharmacological treatments, embryos were bathed in embryo medium with 0.01% DMSO (Thermo Fisher Scientific, Waltham, MA, USA) as vehicle control or 1 μM talarozole (HY-14531, MedChem Express, Monmouth Junction, NJ, USA).

### *In situ* hybridization and immunohistochemistry

Probes for *cyp26b1* [13] and *xirp2a* (cb1045, [2]) are described elsewhere. Color development for fluorescence imaging was performed with α-Digoxigenin-POD F_ab_ fragments (11207733910, Roche Diagnostics, Indianapolis, IN, USA) and TSA Plus Fluorescein (NEL741001KT) or Cy3 (NEL744001KT, Perkin-Elmer, Inc., Waltham, MA, USA) System. In preparation for immunohistochemistry, embryos were fixed in 95% methanol/5% glacial acetic acid. The protocol for myosin heavy chain/Tsp4b staining is described previously [11]. Primary antibodies utilized include MF 20 (1:100 dilution; Developmental Studies Hybridoma Bank, Iowa City, IA, USA), α-Thbs4b (1:200 dilution; GTX125869, GeneTex, Inc., Irvine, CA, USA), α-GFP (1:200 dilution; sc-9996, Santa Cruz Biotechnology, Inc., Dallas, TX, USA). Alexa Fluor-conjugated secondary antibodies from Thermo Fisher Scientific (Waltham, MA, USA) were used at a 1:1000 dilution.

### Microscopy and figure processing

Confocal *z*-stacks were collected using a Zeiss LSM 710 and ZEN software. *In situ* hybridization images were collected using a Zeiss Axio Imager.A1 equipped with an AxioCam HRc, which was operated through AxioVision release 4.9.1 SP1. Images were processed and measured in Fiji [36, 37]. Figures were assembled in the GNU Image Manipulation Program (GIMP).

### Cell counting

Fixed 60 hpf embryos from an incross of *scxa*:*mCherry;cyp26b1*^*b1024*^ were stained with TO-PRO-3 Iodide (642/661) (T3605, Life Technologies, Carlsbad, CA, USA) and imaged as described. Confocal z-stacks were processed and analyzed using Imaris v.8.4.0. Briefly, the Spots module was used to detect nuclei in the TO-PRO channel with a diameter of 3 μm and an automated quality filter and a minimum intensity filter. The Surface module was used to generate a volume rendering of tenoblasts, and a region was selected containing the tenoblasts surrounding the intermandibularis muscles, extending from the oral ectoderm to the point best separating anterior tenoblasts from sternohyoideus tendon condensations. Then, the Imaris XTension “Spots Split to Surface Objects” automatically counted the nuclei located inside the anterior tenoblast Surface object.

### Cell transplantations

Transplantation experiments targeting cranial neural crest were performed as described elsewhere [38, 39].

### Morpholino injection

A previously described morpholino, Cyp26b1-SDEx3 MO [13], was used. Approximately 3 nl of morpholino (working concentration 0.9 mM) were injected into zebrafish embryos between the one-cell and four-cell stages. This concentration of morpholino fully recapitulated the musculoskeletal defects in *b1024* (see Figs 3 and 6).

## Supporting information

S1 Movie Time-lapse confocal projection of cranial muscles and neural crest cells in wild-type and *cyp26b1* mutant embryos. (MOV)

S2 Movie Time-lapse confocal projection of cranial muscles and tenoblasts in control and morpholino-injected (*cyp26b1* MO) embryos. (MOV)

S1 Fig Muscle defects in *cyp26b1* mutants are specific to the ventral midline. (A,B) Dorsal head musculature appears normal in *cyp26b1* mutants (B). am, adductor mandibulae; lap, levator arcus palantini; do, dilator operculi; ao, adductor opercula.
(TIF)

S2 Fig Neural crest mesenchyme surrounds and connects muscles at the mandibulohyoid junction. (Center) 4x digital zoom of muscles and neural crest at the mandibulohyoid junction in a 60 hpf embryo (ventral view, anterior to the left). Orthogonal sections just anterior (A) and posterior (P) of the mandibulohyoid junction show slices through both intermandibularis posterior muscles or both interhyal muscles, respectively. Orthogonal sections to the left (L) and right (R) of the mandibulohyoid junction show slices through connections between intermandibularis posterior and interhyal muscles. In each slice, mesenchymal neural crest cells can be seen filling the space between the muscles and surrounding the muscles. Scale bar = 50μm.
(TIF)

S3 Fig The mandibulohyoid junction is free of *scxa;sox9a* dual positive progenitors. (A-A”) Projection of the ventral pharyngeal arches of a *scxa*:*mCherry;sox9a*:*EGFP* double transgenic zebrafish. (B-B”) Single z-slice of the same fish at higher magnification. While there are *scxa;sox9a* double positive cells at the tips of the sternohyoideus tendon (B, arrowhead), none are apparent in the basihyal which is immediately dorsal to the mandibulohyoid junction.
(TIF)

S4 Fig Defects to ceratohyal symphysis formation correspond with mispatterning of tenoblasts. (A,E) Confocal images from Fig 6A and 6B are projected to show tenoblasts and *fli1*:*egfp*-positive neural crest cells. B-D and F-H are slices through A and E, respectively. Red arrows indicate intersections of muscle tips and tenoblasts. Dashed lines in B and C show the plane of the orthogonal slice in D, and F-H follow the same convention. (B) Just posterior to condensing tenoblasts at the mandibulohyoid junction, an elongated group of *fli1*:*EGFP* exprssing cells fits between the ceratohyal cartilage condensations (arrowhead). (C) At their dorsal/posterior end, these cells separate the two sternohyoideus tendon condensations (arrowhead). (F) In *cyp26b1* mutants we see elongated and bright GFP-positive cells, but their morphology is disrupted (open green arrowhead). An ellipsoid group of these cells sits between the anterior and posterior tenoblast populations, but those tenoblast populations are not segregated (asterisk in F). (G) Dorsally, no neural crest cells extend between the sternohyoideus tendon condensations, and tenoblasts appear to reach between the ceratohyal cartilages toward the mandibulohyoid junction (solid green arrowhead). We used orthogonal sections to understand the arrangement of cells in the midline across the anterior-posterior width of the second pharyngeal arch. (D) Posterior tenoblasts are present in the dorsal/posterior quadrant of the midline at 60 hpf in wild-type embryos, and mandibulohyoid junction tenoblasts reside in the ventral/anterior quadrant. (H) In *cyp26b1* mutants, tenoblasts overextend the mandibulohyoid junction (open green arrowhead) and also fill the space between ceratohyal cartilages (solid green arrowhead) to occupy all four quadrants in the midline. All images ventral view, anterior to the left. Scale bar = 50μm.
(TIF)

S5 Fig Tenoblasts populate the mandibulohyoid junction prior to muscle attachment. (A) Tenoblasts surround muscles as early as 54 hpf. (B) By 57 hpf, tenoblasts are present in the midline, medial to the location of the muscle fibers.
(TIF)

S6 Fig Intermandibularis posterior muscle elongation toward the midline is inhibited by loss of Cyp26b1 function. At 51 hpf, bilateral intermandibularis muscle masses elongate along the surface of the first pharyngeal arch in control (A) and *cyp26b1*-depleted embryos (C). (B) In control embryos, the intermandibularis posterior muscles point their posterior tips toward the midline at 54 hpf, roughly perpendicular to the initial muscle masses (arrows). (D) The intermandibularis posterior muscles point posteriorly at 54 hpf, almost parallel to each other (arrows). All images ventral view, anterior to the left. Scale bar = 50μm.
(TIF)

S7 Fig Expression of *cyp26b1* is not restricted to ventral mesenchyme. A single confocal z-slice showing that the tip of the adductor mandibulae (am, labeled with MF20 antibody in red) is adjacent to *cyp26b1* expressing cells (blue). The expression of *cyp26b1* appears to be in non-*scxa*-expressing cells (green).
(TIF)

S8 Fig A tenoblast non-autonomous requirement for *cyp26b1*. (A,B) Small groups of cells were transplanted from *scxa*:*mCherry;fli1*:*EGFP* double transgenic embryo into embryos from crosses between *cyp26b1* carriers. Embryos were imaged at 54 hpf to identify embryos in which the transplanted cells contributed to only the *scxa*-lineage within the ventral 2^nd^ pharyngeal arch (insets, asterisks, oral opening; arrowhead, tenoblasts). Fish were grown to 4 dpf, stained for myosin via MF20 and reimaged. Cells contributing to the ventral arches in wild-type embryos formed elongated processes at the mandibulohyoid junction (A, arrowhead) and associated closely with the muscle fibers. (B) Wild-type tenoblasts transplanted into a *cyp26b1* mutant fail to form cell processes at the mandibulohyoid junction (arrowhead) or associate closely with the musculature (arrow).
(TIF)
