# Supplementary figures and images for: In vivo zebrafish morphogenesis shows Cyp26b1 promotes tendon condensation and musculoskeletal patterning in the embryonic jaw
Source: PLoS Genet. 2017 Dec 11;13(12):e1007112. doi: 10.1371/journal.pgen.1007112 (PMC5739505; doi:10.1371/journal.pgen.1007112)

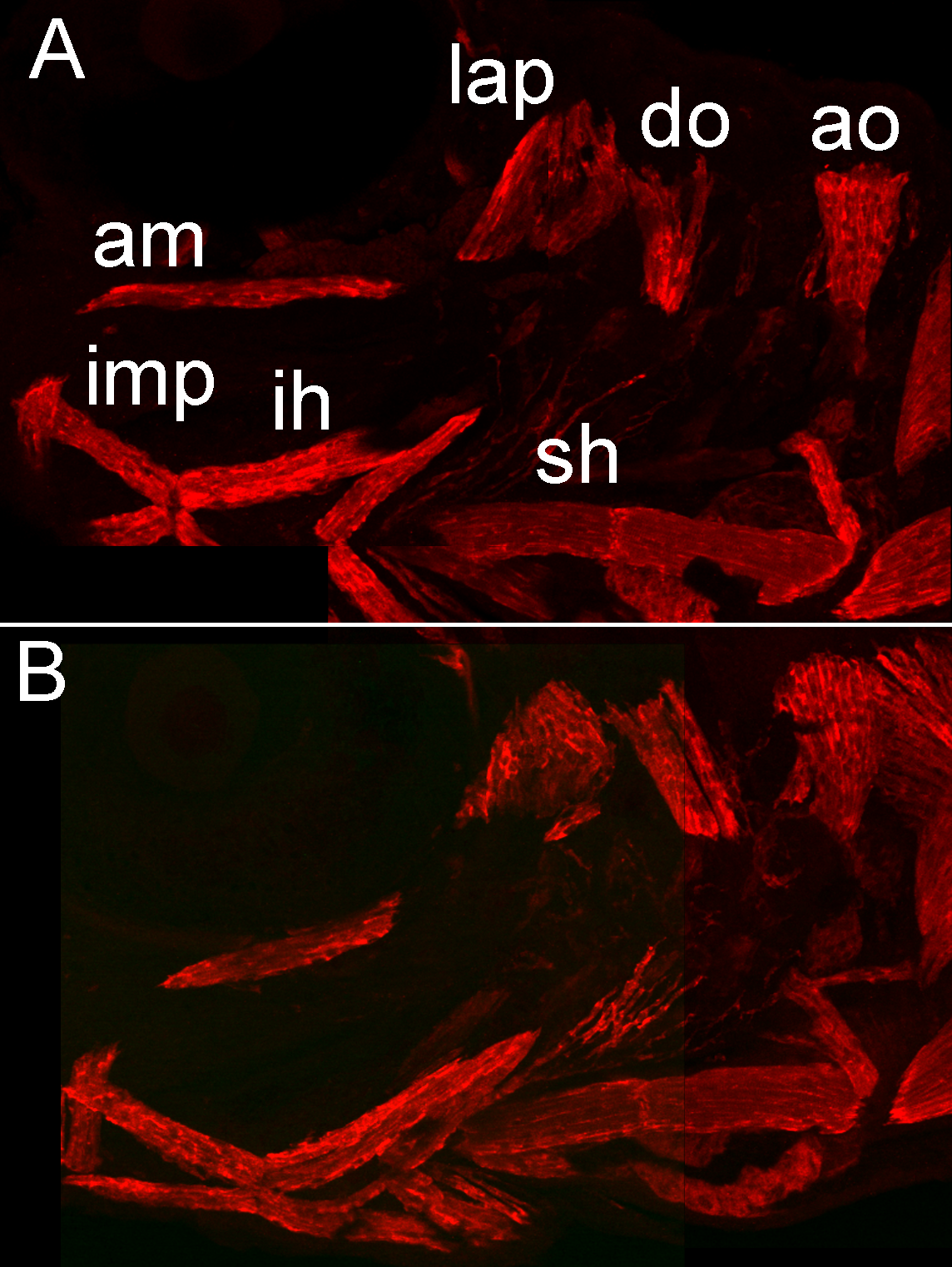

Supplement: S1 Fig — (A,B) Dorsal head musculature appears normal in cyp26b1 mutants (B). am, adductor mandibulae; lap, levator arcus palantini; do, dilator operculi; ao, adductor opercula. (TIF) [file pgen.1007112.s003.tif]

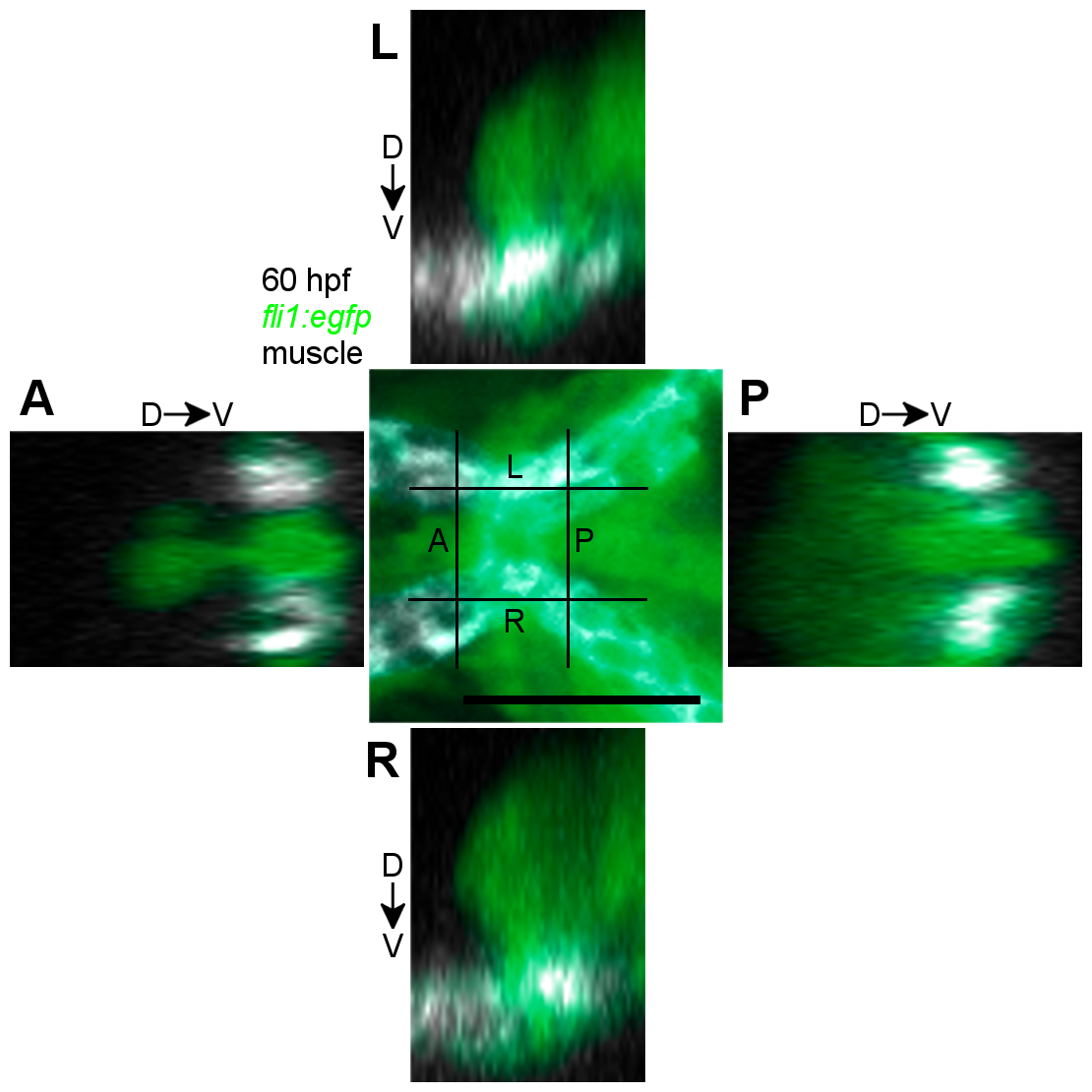

Supplement: S2 Fig — (Center) 4x digital zoom of muscles and neural crest at the mandibulohyoid junction in a 60 hpf embryo (ventral view, anterior to the left). Orthogonal sections just anterior (A) and posterior (P) of the mandibulohyoid junction show slices through both intermandibularis posterior muscles or both interhyal muscles, respectively. Orthogonal sections to the left (L) and right (R) of the mandibulohyoid junction show slices through connections between intermandibularis posterior and interhyal muscles. In each slice, mesenchymal neural crest cells can be seen filling the space between the muscles and surrounding the muscles. Scale bar = 50μm. (TIF) [file pgen.1007112.s004.tif]

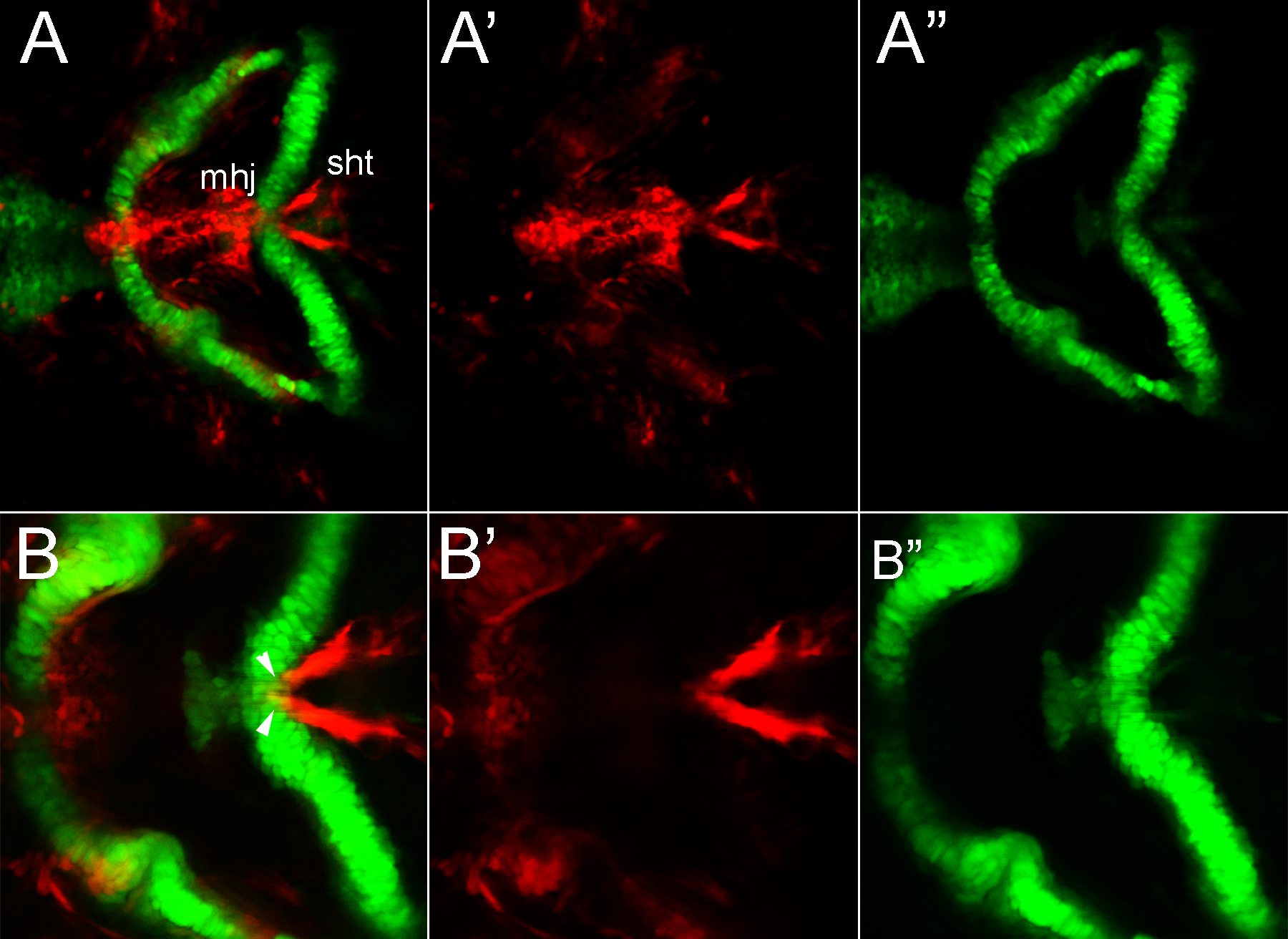

Supplement: S3 Fig — (A-A”) Projection of the ventral pharyngeal arches of a scxa:mCherry;sox9a:EGFP double transgenic zebrafish. (B-B”) Single z-slice of the same fish at higher magnification. While there are scxa;sox9a double positive cells at the tips of the sternohyoideus tendon (B, arrowhead), none are apparent in the basihyal which is immediately dorsal to the mandibulohyoid junction. (TIF) [file pgen.1007112.s005.tif]

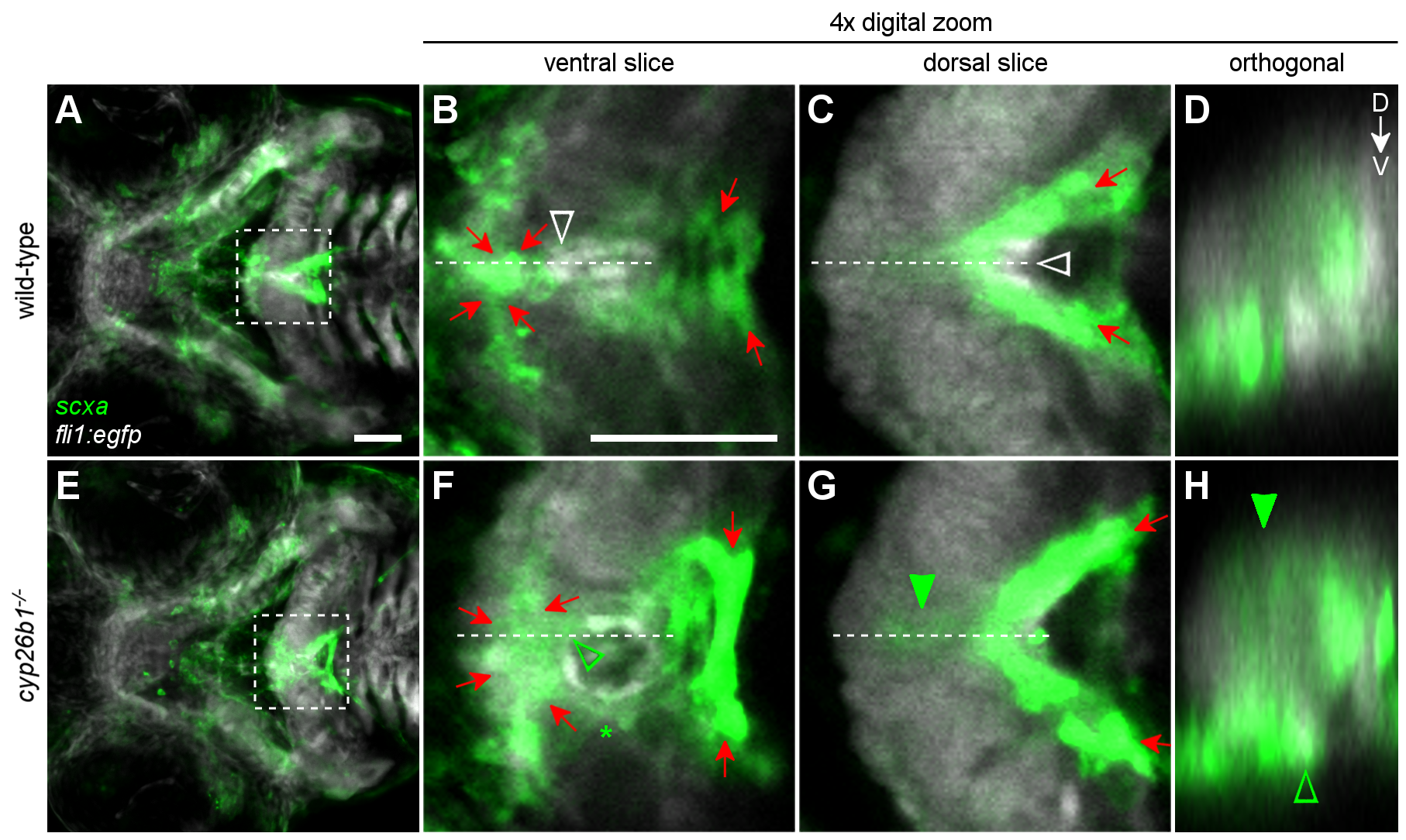

Supplement: S4 Fig — (A,E) Confocal images from Fig 6A and 6B are projected to show tenoblasts and fli1:egfp-positive neural crest cells. B-D and F-H are slices through A and E, respectively. Red arrows indicate intersections of muscle tips and tenoblasts. Dashed lines in B and C show the plane of the orthogonal slice in D, and F-H follow the same convention. (B) Just posterior to condensing tenoblasts at the mandibulohyoid junction, an elongated group of fli1:EGFP exprssing cells fits between the ceratohyal cartilage condensations (arrowhead). (C) At their dorsal/posterior end, these cells separate the two sternohyoideus tendon condensations (arrowhead). (F) In cyp26b1 mutants we see elongated and bright GFP-positive cells, but their morphology is disrupted (open green arrowhead). An ellipsoid group of these cells sits between the anterior and posterior tenoblast populations, but those tenoblast populations are not segregated (asterisk in F). (G) Dorsally, no neural crest cells extend between the sternohyoideus tendon condensations, and tenoblasts appear to reach between the ceratohyal cartilages toward the mandibulohyoid junction (solid green arrowhead). We used orthogonal sections to understand the arrangement of cells in the midline across the anterior-posterior width of the second pharyngeal arch. (D) Posterior tenoblasts are present in the dorsal/posterior quadrant of the midline at 60 hpf in wild-type embryos, and mandibulohyoid junction tenoblasts reside in the ventral/anterior quadrant. (H) In cyp26b1 mutants, tenoblasts overextend the mandibulohyoid junction (open green arrowhead) and also fill the space between ceratohyal cartilages (solid green arrowhead) to occupy all four quadrants in the midline. All images ventral view, anterior to the left. Scale bar = 50μm. (TIF) [file pgen.1007112.s006.tif]

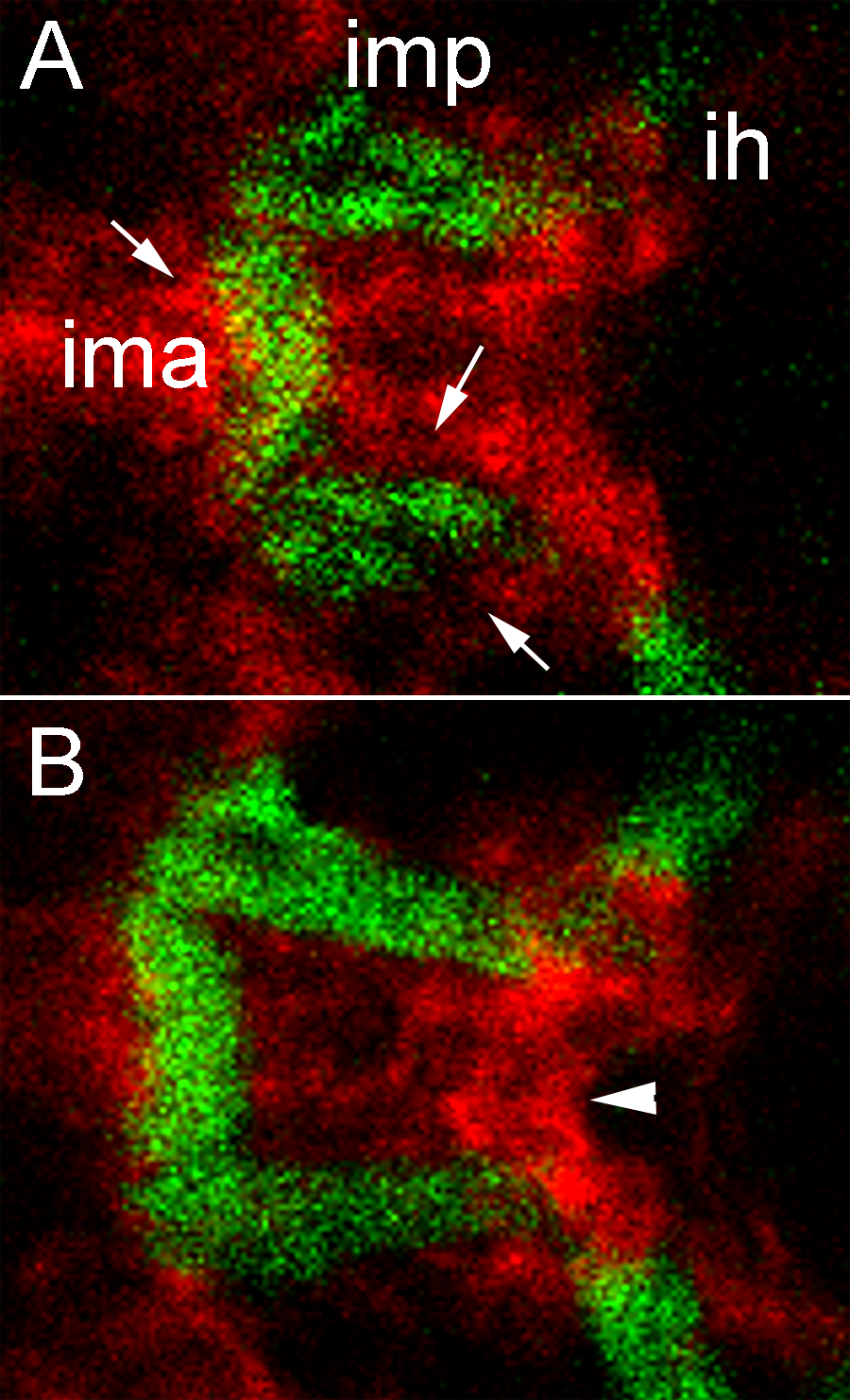

Supplement: S5 Fig — (A) Tenoblasts surround muscles as early as 54 hpf. (B) By 57 hpf, tenoblasts are present in the midline, medial to the location of the muscle fibers. (TIF) [file pgen.1007112.s007.tif]

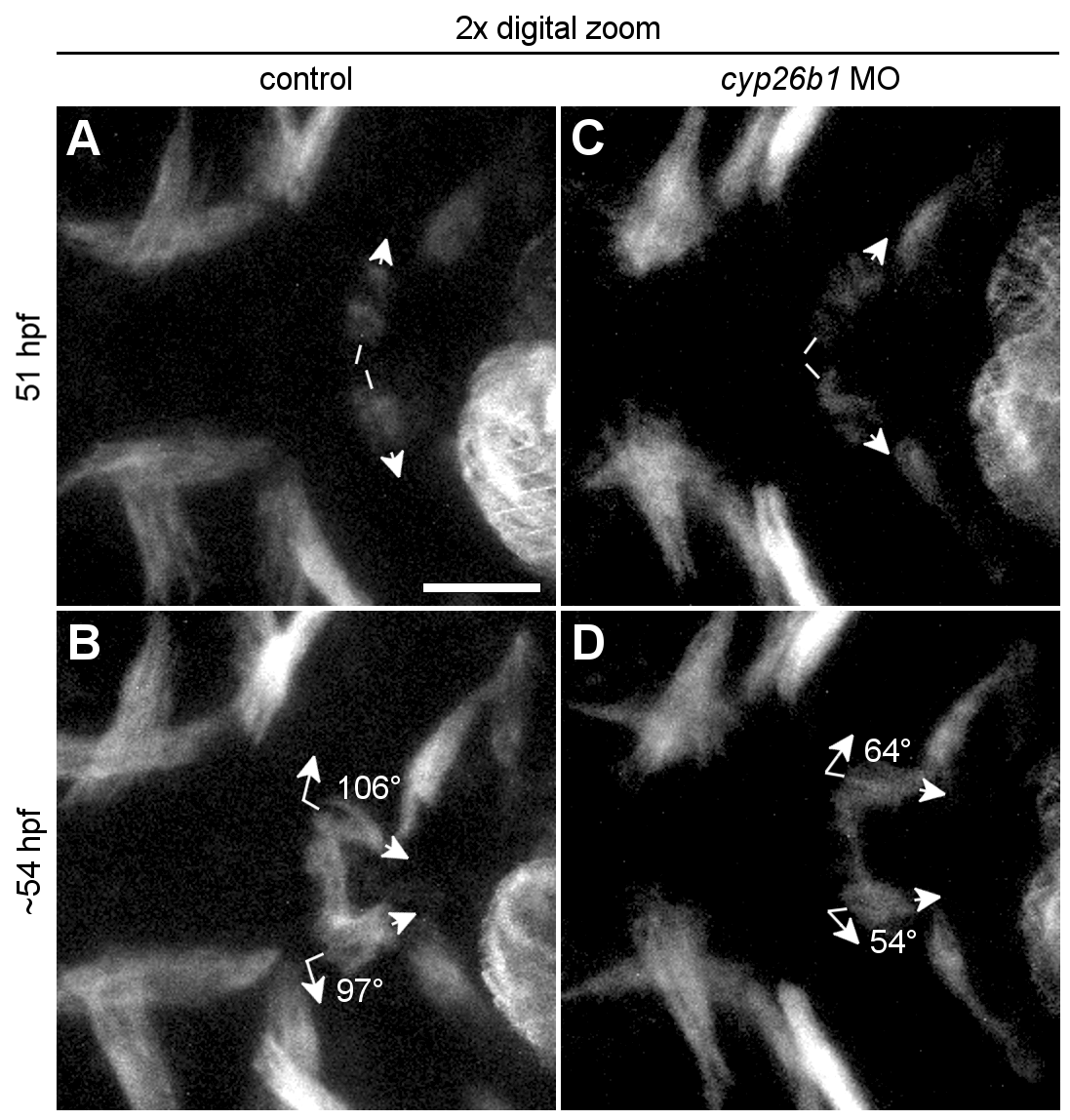

Supplement: S6 Fig — At 51 hpf, bilateral intermandibularis muscle masses elongate along the surface of the first pharyngeal arch in control (A) and cyp26b1-depleted embryos (C). (B) In control embryos, the intermandibularis posterior muscles point their posterior tips toward the midline at 54 hpf, roughly perpendicular to the initial muscle masses (arrows). (D) The intermandibularis posterior muscles point posteriorly at 54 hpf, almost parallel to each other (arrows). All images ventral view, anterior to the left. Scale bar = 50μm. (TIF) [file pgen.1007112.s008.tif]

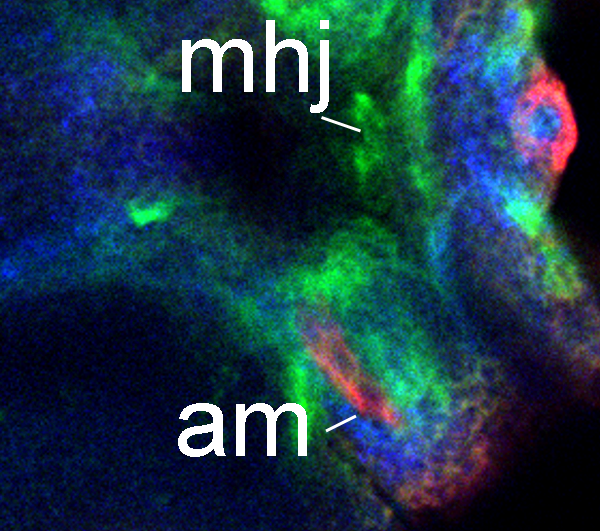

Supplement: S7 Fig — A single confocal z-slice showing that the tip of the adductor mandibulae (am, labeled with MF20 antibody in red) is adjacent to cyp26b1 expressing cells (blue). The expression of cyp26b1 appears to be in non-scxa-expressing cells (green). (TIF) [file pgen.1007112.s009.tif]

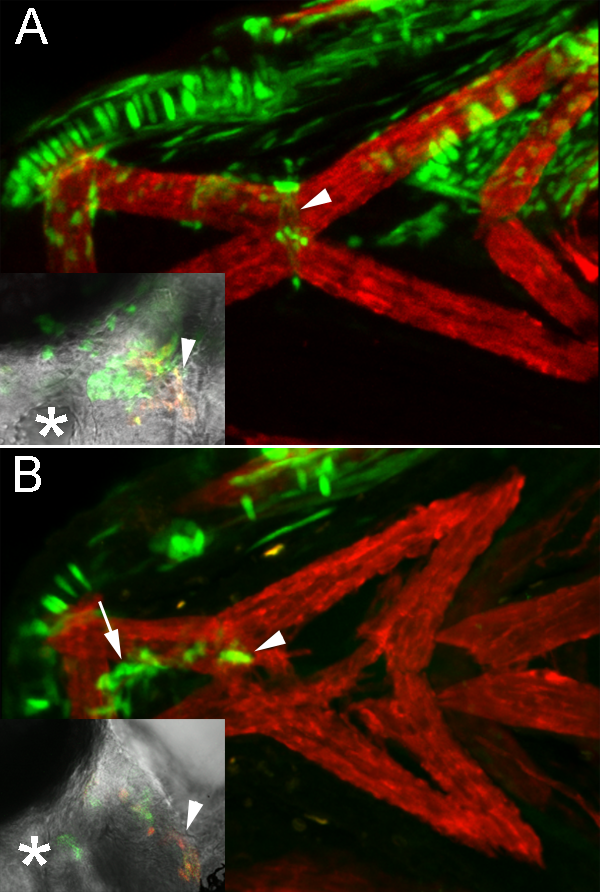

Supplement: S8 Fig — (A,B) Small groups of cells were transplanted from scxa:mCherry;fli1:EGFP double transgenic embryo into embryos from crosses between cyp26b1 carriers. Embryos were imaged at 54 hpf to identify embryos in which the transplanted cells contributed to only the scxa-lineage within the ventral 2nd pharyngeal arch (insets, asterisks, oral opening; arrowhead, tenoblasts). Fish were grown to 4 dpf, stained for myosin via MF20 and reimaged. Cells contributing to the ventral arches in wild-type embryos formed elongated processes at the mandibulohyoid junction (A, arrowhead) and associated closely with the muscle fibers. (B) Wild-type tenoblasts transplanted into a cyp26b1 mutant fail to form cell processes at the mandibulohyoid junction (arrowhead) or associate closely with the musculature (arrow). (TIF) [file pgen.1007112.s010.tif]
